# Context-Dependent Suppression of Thrips by Insect-Proof Nets in Greenhouse Eggplant and Potato

**DOI:** 10.3390/insects17090883

**Published:** 2026-08-24

**Authors:** Lin Yi, Junjie Yan, Shovon Chandra Sarkar, Ziying Wang, Yulin Gao

**Affiliations:** 1 Key Laboratory of Agricultural Biosafety and Green Production of Upper Yangtze River (Ministry of Education), College of Plant Protection, Southwest University, Chongqing 400715, China; 18372667753@163.com; 2 State Key Laboratory for Biology of Plant Diseases and Insect Pests, Institute of Plant Protection, Chinese Academy of Agricultural Sciences, Beijing 100193, China; yanjunjie01@caas.cn; 3 Food Futures Institute, Murdoch University, Perth, WA 6150, Australia; shovon.sarkar@murdoch.edu.cn

**Keywords:** insect-proof net, eggplant, potato, thrips, microclimate, integrated pest management

## Abstract

Insect-proof nets provide a non-chemical alternative within integrated pest management (IPM), but their effectiveness against thrips may depend on crop species, season, mesh size, and treatment-associated microclimate conditions. We compared an uncovered and unframed control (CK) and 40-, 60-, and 80-mesh insect-proof nets on thrip abundance in eggplant and potato across two planting seasons in a single-span plastic greenhouse. Weekly plot-level thrip surveys were conducted using a five-point sampling method. Thrips were not identified to species, and the response variable therefore represented total thrip abundance. Treatment effects were estimated with negative binomial (NB1) generalized linear mixed models incorporating a random intercept for plot and an offset for sampling effort; microclimate variables were analyzed with linear mixed models, and exploratory analyses additionally adjusted the NB1 model for measured microclimate covariates to examine whether accounting for these variables altered the estimated net-cage treatment associations. Net-cage treatments generally reduced thrip abundance relative to the control, but the magnitude and consistency of suppression varied markedly with crop and season. The 60-mesh net showed the most consistent direction of suppression, with estimated reductions ranging from 36.0% to 44.4% across the four crop–season contexts; this suppression was statistically supported in both eggplant contexts, but only marginal in the two potato contexts, and this directional consistency did not imply universal superiority in all contexts. The 80-mesh net produced stronger suppression in some cases but was inconsistent, with no detectable suppression in eggplant during the second season. Treatment-related differences in 7-day temperature and relative humidity were generally statistically uncertain, whereas illuminance showed some treatment-associated variation. Adjustment for the measured microclimate covariates did not consistently attenuate the estimated treatment associations, indicating that the measured variables did not provide a simple statistical explanation for the observed context-dependent pattern. Overall, the 60-mesh treatment showed the most consistent estimated suppressive direction under the tested conditions, whereas increasing nominal mesh count did not produce a simple monotonic increase in suppression. The microclimate-adjusted analysis was exploratory and did not provide a causal decomposition of net-cage effects.

## Simple Summary

Thrips are small insect pests that can damage greenhouse vegetable crops and are difficult to manage using chemical control alone. Insect-proof nets are widely used as a physical barrier. We tested 40-, 60-, and 80-mesh insect-proof nets in greenhouse eggplant and potato crops across two planting seasons. Net-cage treatment effects varied markedly among crop–season contexts, indicating that suppression depended on both crop species and planting season. The 60-mesh treatment showed the most consistent estimated direction of suppression across the four crop–season contexts, with reductions ranging from 36.0% to 44.4%. The 80-mesh treatment produced stronger suppression in some contexts but no detectable suppression in eggplant during Season 2. Differences in 7-day temperature and relative humidity were generally uncertain, whereas short-duration illuminance differed among treatments in several contexts. These results indicate that increasing nominal mesh count does not necessarily produce a monotonic increase in thrip suppression.

## 1. Introduction

Thrips (Insecta: Thysanoptera) are among the most destructive global agricultural pests. These small insects use their piercing–sucking mouthparts to penetrate tissues and extract sap from leaves, petals, and fruits, causing leaf curling, wilting, premature petal senescence, and fruit deformation. These symptoms result in reduced fruit quality and yield, thereby lowering the economic value of crops. In eggplants (*Solanum melongena* L.), thrips damage flower buds and fruits, causing poor pollination and a high rate of malformed fruits exceeding 30% [1,2,3]. Thrips reproduce by both parthenogenesis and sexual reproduction; their short generational cycle and relatively long adult stage result in high reproductive capacity and prolonged crop damage, leading to rapid population expansion and exacerbating crop yield losses [4,5,6,7].

Currently, chemical insecticides are the primary method for controlling thrips. However, overreliance on insecticides has facilitated the rapid development of insecticide resistance in thrips, and improper pesticide use has raised concerns about health risks from pesticide residues and environmental pollution [8]. Consequently, environmentally friendly pest management strategies have attracted increasing attention. As a core physical control, insect-proof nets can effectively block thrips by creating a physical barrier. Nonetheless, nets with lower mesh counts are less effective against the tiny-sized thrips. Higher mesh count nets, which have smaller pores, can significantly impede airflow and affect ventilation, leading to excessive temperature and humidity inside the net [9].

Temperature and humidity can influence thrips’ development, survival, reproduction, activity, and population dynamics, but the direction and magnitude of these responses depend on species, developmental stage, and the environmental range experienced. For example, laboratory studies of *Frankliniella occidentalis* have reported reduced development and survival under extremely high-temperature conditions [10,11,12]. Because thrips in the present experiment were not identified to species, such species-specific findings are cited only as biological background and are not assumed to characterize the sampled field population. Some studies have reported positive associations between relative humidity and thrip survival or population development, but such relationships should not be assumed to apply uniformly across species or greenhouse conditions [13,14,15]. Changes in greenhouse temperature, humidity, and radiation may also influence crop performance and management requirements [9].

Previous studies have established that insect-proof nets can both restrict pest movement and modify the protected crop’s microclimate [16,17]. Therefore, the novelty of the present study does not lie in proposing these two pathways. Rather, our study addresses a narrower question: whether the magnitude and consistency of net-cage-associated thrips suppression vary among crop–season contexts, and whether those context-specific treatment estimates remain stable after statistical adjustment for the measured microclimate variables. Previous pest-monitoring studies have used temporal or forecasting models to characterize within- and between-season population dynamics [18,19], whereas our primary analysis uses a repeated-measures negative binomial mixed model to estimate treatment-to-control incidence rate ratios within Crop × Season contexts. We then refit the model with measured temperature, relative humidity, and illuminance as additional covariates solely as a sensitivity analysis. This comparison is intended to assess the robustness of the estimated treatment associations rather than to predict future thrip abundance or partition the treatment effect into causal physical and microclimatic pathways.

In our study, we evaluated three insect-proof nets (minimum pore widths ≈ 0.922 mm, 0.495 mm, and 0.300 mm) against an unmeshed control in eggplant and potato over two planting seasons in the same greenhouse. Given that the thorax and abdomen widths of thrips are approximately 0.251 mm and 0.271 mm, none of the mesh apertures were smaller than the thrips’ body dimensions, making complete physical exclusion unlikely for any net. We hypothesized that finer meshes would impose greater restriction on thrips’ movement or entry, but that the resulting suppression would depend on crop and season because host plant traits and microclimate also influence thrips populations. To capture this context dependence, we estimated net-cage treatment effects separately for each Crop × Season combination using repeated weekly observations and negative binomial mixed models, and we compared the direction and consistency of these effects across the four contexts. Based on the same set of 307 plot-week observations, we further compared unadjusted and microclimate-adjusted estimates as an exploratory sensitivity analysis, not to partition direct physical and indirect microclimate effects, but to assess the robustness of the treatment associations to adjustment for measured temperature, relative humidity, and illuminance. Our results indicate that increasing mesh count did not lead to a monotonic increase in thrips suppression, and that the measured microclimate variables could not provide a simple explanation for the context-dependent variation in treatment effects.

The objectives of our study were to: (1) characterize the physical properties of the three insect-proof nets; (2) quantify net-cage treatment effects on thrip abundance and determine how their magnitude and consistency varied among crop–season contexts; and (3) evaluate whether treatment-associated differences in the measured microclimate variables were related to variation in thrip suppression and could provide a simple statistical explanation for the observed context dependence.

## 2. Materials and Methods

### 2.1. Study Site and Experimental Design

**Figure 1:**
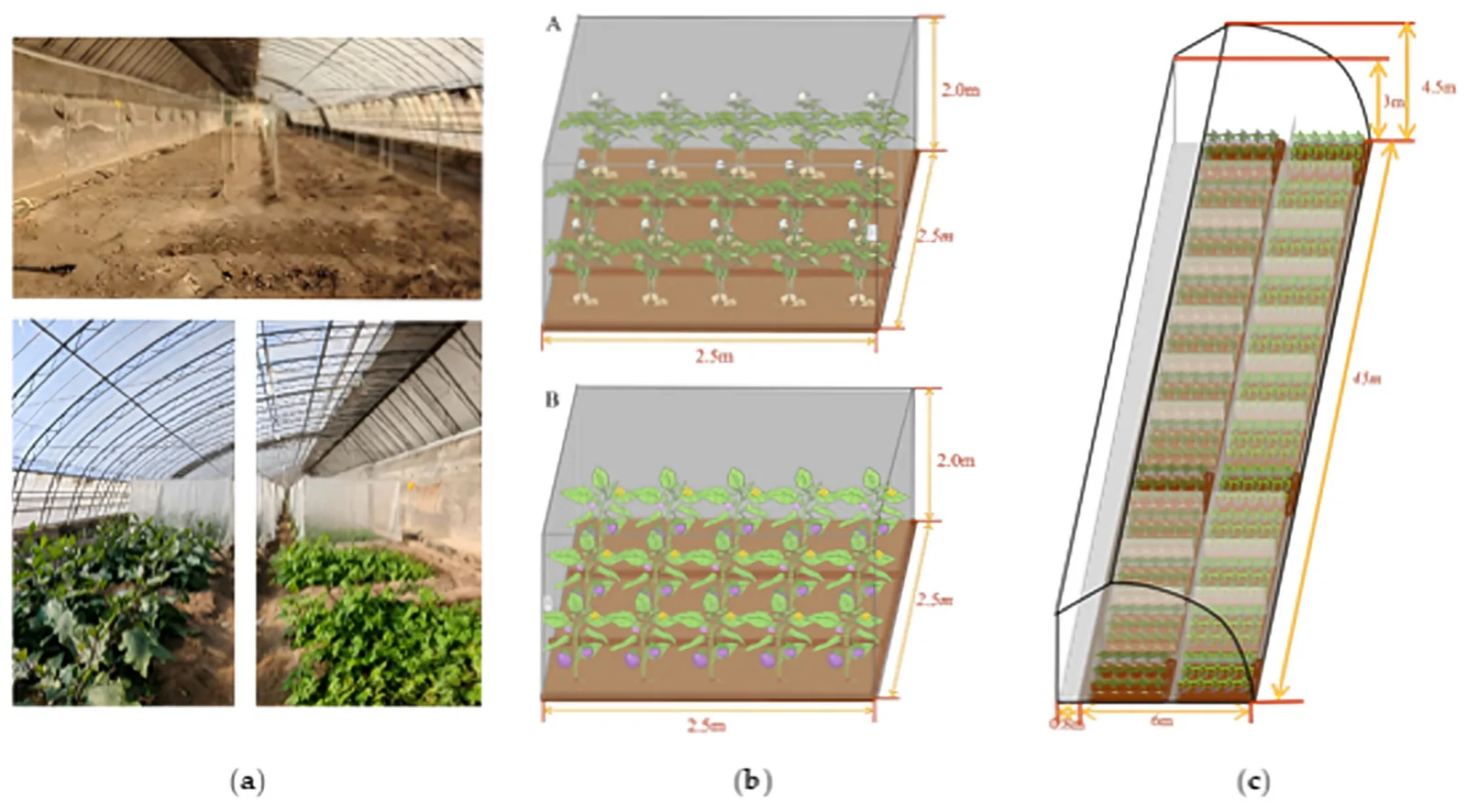
Schematic illustration of the experimental greenhouse and crop planting layout. (**a**) Interior view of the experimental greenhouse and crop establishment; (**b**) planting arrangement within an individual experimental plot, where (**A**) indicates the potato plot layout and (**B**) indicates the eggplant plot layout; (**c**) overall planting layout of the greenhouse experiment.

We conducted the experiment in a single-span plastic greenhouse at the Langfang Experimental Station of the Chinese Academy of Agricultural Sciences, Hebei Province, China (116°36′26″ E, 39°30′39″ N). We used a greenhouse oriented in a north–south direction and covered with clear polyethylene film. Natural ventilation was provided through side vents located along the north side of the greenhouse, and vent openings were manually adjusted according to crop growth stage and weather conditions. We present detailed structural parameters of the greenhouse in Figure 1.

We used two crops: potato and eggplant. Four net-cage treatments were established: an uncovered control (CK) with no netting and no frame, and three insect-proof nets of 40-mesh, 60-mesh, and 80-mesh (white, polyethylene). The experiment was arranged as a randomized complete block design (RCBD). For each crop, three blocks were established, and each block contained four experimental plots. The four net-cage treatments (CK, 40-mesh, 60-mesh, and 80-mesh) were randomly assigned to the four plots within each block, so that each treatment occurred once in each block. Thus, each treatment was represented by three replicate plots per crop, giving 12 plots per crop and 24 plots per planting season. The individual plot was the experimental unit for the net-cage treatment.

Block was recorded as a numerical identifier for the randomized block, whereas PlotID uniquely identified each experimental plot. Weekly observations were repeatedly collected from the same plots throughout each growing season. A season-specific PlotID was therefore used to link repeated observations from the same experimental unit. Each plot measured 2.5 m × 2.5 m (6.25 m^2^) and was separated from neighboring plots by a 1 m buffer zone. Plots with the same crop were grouped together in an east–west orientation.

In the net-cage treatments, a cubic frame (2.5 m × 2.5 m × 2 m) of steel pipes was erected over the plot and completely covered with the assigned netting; one side was fitted with a 2 m-high zippered opening to allow field management and sampling. Consequently, comparisons between CK and the net-cage treatments represent differences associated with the complete net-cage system rather than effects attributable solely to the mesh material. The mesh size of the insect-proof nets was defined as the number of openings per square inch (2.54 cm × 2.54 cm), ensuring standardized classification of net-cage treatments across the experiment. A unique season-specific PlotID was assigned to each experimental plot, yielding 24 PlotIDs per season and 48 PlotIDs across the two seasons. Repeated weekly observations within each season-specific plot were linked by PlotID.

In 2025, we conducted two planting seasons: the spring season from February to May and the autumn season from September to December. These two periods were designed to capture seasonal variation in greenhouse microclimate and pest dynamics under different insect-proof net-cage treatments.

For potato, we used the cultivar “Marko”, planted seed tubers with approximately 1 cm sprouts at a density of 2.4 plants m^−2^, and installed insect-proof nets one week after seedling emergence to ensure initial establishment before exposure to treatment conditions.

For eggplant, we selected the cultivar “Heibao Yuanqie”, transplanted seedlings at a density of 2.4 plants m^−2^ and applied insect-proof nets when plants reached the 7–8 true leaf stage, ensuring that plants had fully recovered from transplanting stress before the treatments were applied.

We monitored the experiment over two distinct periods in 2025. Season 1 covered weeks 15–20 (spring planting), and Season 2 covered weeks 42–52 (autumn planting). During both seasons, we sampled thrips weekly, measured illuminance weekly and recorded temperature and relative humidity hourly.

For modeling purposes, the four crop–season combinations were combined into a single factor, Context, with four levels: eggplant S1, eggplant S2, potato S1, potato S2.

### 2.2. Measurement of Insect-Proof Net Characteristics and Thrips’ Body Dimensions

The insect-proof nets used in this experiment were white and manufactured from polyethylene by filament drawing and weaving; therefore, net color was held constant across treatments and was not evaluated as an experimental factor in the present study. The aperture dimensions and filament diameters were measured using an Olympus SZX16 stereo microscope (type: SZX16; Shanghai Pooher Laboratory Technology Co., Ltd., Shanghai, China) coupled to a digital camera. Images were initially captured via the Image View software (version: x64, 4.10.17659.20200906) and subsequently transferred to ImageJ (version: 1.46r) for length measurements. The microscopic images were transmitted through a video interface from the camera to the software, allowing subsequent dimensional analysis in ImageJ.

To compare thrips’ body size with the aperture dimensions of insect-proof nets, 50 adult thrips collected from the greenhouse were randomly selected and measured under a stereo microscope. The widest parts of the thorax and abdomen were measured. All measurements were performed using ImageJ software after scale calibration.

For each mesh specification, ten 10 cm × 10 cm pieces were cut from different positions of the net. One aperture was randomly selected from each piece, and its horizontal aperture dimension (*A_i_*), vertical aperture dimension (*B_i_*), and horizontal filament diameter (*d_i_*) were measured. Thus, 10 measurements were obtained for each mesh specification. Porosity was calculated separately for each net piece as follows:

$$P_{i}=\frac{A_{i}\times B_{i}}{\left(A_{i}+d_{i})(B_{i}+d_{i}\right)}\times 100\%$$

where *P_i_* is the porosity of the *i*th net piece, *A_i_* and *B_i_* are its horizontal and vertical aperture dimensions, respectively, and *d_i_* is its measured horizontal filament diameter used as the representative filament diameter. The mean and standard deviation of the 10 calculated porosity values were then reported for each mesh specification.

### 2.3. Thrip Sampling

Thrip abundance was surveyed weekly using a five-point sampling design within each plot [20]. At each sampling point, one fully expanded leaf was randomly selected from the middle canopy, with canopy position defined relative to plant height rather than a fixed distance above the ground. A white A4 paper sheet was placed beneath the leaf, and thrips were dislodged by gently tapping the leaf and counted immediately. All surveys were conducted between 09:00 and 11:00 on the same day of the week by a single trained observer. The same sampling procedure, canopy-position criterion, survey timing, and observer were used for both eggplant and potato. Thrips were not differentiated by species or developmental stage. The total number recorded in each plot on each sampling occasion was defined as Thrips_total, and the number of valid sampling points was recorded as n_thrips_points and incorporated into the count model as an offset.

### 2.4. Microclimate Measurements

We recorded air temperature and relative humidity inside each plot using Elitch RC-4HC data loggers (type: RC-4HC; Jiangsu Jingchuang Electronics CO., Ltd., Xuzhou, China). One logger was placed at the center of each plot, mounted at a fixed height of 1.5 m above the ground, and this height was not altered as the crop grew. No radiation shield was used. Loggers recorded data at hourly intervals throughout each growing season. For each thrip sampling date, we calculated the mean, maximum, and minimum hourly temperature and relative humidity over the preceding 7 days. The 7-day means were retained as the microclimate variables in the linear mixed models and the exploratory microclimate-adjusted thrip model, whereas the corresponding weekly maxima and minima were summarized descriptively to characterize short-term environmental extremes. The manufacturer stated accuracy for the loggers is ±0.5 °C for temperature (−20 to 40 °C) and ±1 °C outside this range, and ±3% RH (at 25 °C, 20–80% RH) and ±5% RH otherwise. All loggers received supplier calibration before the experiment; no independent calibration was performed by the authors.

We measured illuminance weekly immediately after the thrip survey, using a digital lux meter (type: PP730; Beijing Jiuyan Technology Co., Ltd., Beijing, China), and took measurements at the center of each plot at approximately 12:00 noon local time. The meter was held horizontally at 1.5 m height, and the mean illuminance over an approximately 30 s measurement period was recorded. These data represent a short-duration photometric measurement and are referred to as illuminance; they are not photosynthetically active radiation (PAR) and should not be interpreted as weekly mean light intensity.

### 2.5. Statistical Analysis

We conducted all statistical analyses in R software (version 4.5.0, R Core Team, Vienna, Austria). We converted categorical variables, including Net, Context, Block, and PlotID, into factors. Relative sampling week was standardized before model fitting. Temperature, relative humidity, and illuminance were additionally standardized when used as covariates in the exploratory microclimate-adjusted thrips model.

#### 2.5.1. Primary Model for Thrip Abundance

Let *Y_ij_* denote the observed thrip count for the *i*th observation (weekly survey) in plot *j* (PlotID). We modeled *Y_ij_* using a negative binomial generalized linear mixed model with a linear mean–variance parameterization (NB1):

$$Y_{ij}∼\mathrm{NegBin}(\mu_{ij},\phi )$$

$$\log(\mu_{ij})=\beta_{0}+\beta_{\mathrm{Net}[i]}+\beta_{\mathrm{Context}[i]}+\beta_{\mathrm{Net}\times \mathrm{Context}[i]}+\beta_{w}{\text{Week\_z}}_{ij}+\log({\text{n\_thrips\_points}}_{ij})+b_{j}$$

*β*_Net[*i*]_ are the fixed effects for the four net-cage treatments (CK, 40-mesh, 60-mesh, 80-mesh); *β*_Context[*i*]_ are the fixed effects for the four crop–season contexts; *β*_Net×Context[*i*]_ are the interaction effects; *β_w_* is the fixed slope for standardized sampling week (Week_z); $\log({\text{n\_thrips\_points}}_{ij})$ is an offset term (coefficient fixed to 1) that adjusts for unequal sampling effort; $b_{j}∼N(0,\sigma_{plot}^{2})$ is a random intercept for plot, accounting for repeated weekly observations within the same plot; *ϕ* is the dispersion parameter of the NB1 distribution, where Var (*Y_ij_*) = *μ_ij_* + *ϕμ_ij_*.

From this model, treatment contrasts (each net treatment vs. CK) were obtained using the emmeans package and expressed as incidence rate ratios (IRR) with 95% confidence intervals. The percent reduction in thrip abundance relative to CK was calculated as (1 − IRR) × 100%.

Because repeated weekly observations were obtained from the same experimental plots, PlotID was included as a random intercept in the primary model to account for within-plot correlation across sampling weeks. Block and PlotID represented different components of the experimental design: Block represented the randomized blocking structure, whereas PlotID represented the experimental unit on which repeated measurements were obtained.

To evaluate whether explicit adjustment for the randomized block structure materially affected the treatment estimates, we performed an additional sensitivity analysis. Because only three blocks were available within each Crop × Season context, block was entered as a fixed design-control factor nested within Crop × Season rather than as an additional random-effect variance component. The sensitivity model therefore retained the same NB1 distribution, fixed treatment structure, standardized week covariate, sampling-effort offset, and PlotID random intercept as the primary model, with the addition of the context-specific block term. Robustness was evaluated by comparing context-specific Net-versus-CK IRRs, 95% confidence intervals, effect directions, and *p* values between the primary and block-adjusted models.

$$\begin{array}{cc} \text{Thrips\_total} & ∼\mathrm{Poisson}(\mu )\\ \log(\mu ) & =\beta_{0}+\beta_{\mathrm{Net}}+\beta_{\mathrm{Crop}}+\beta_{\mathrm{Season}}\\ & \;+\;\beta_{\mathrm{Net}:\mathrm{Crop}}+\beta_{\mathrm{Net}:\mathrm{Season}}+\beta_{\mathrm{Crop}:\mathrm{Season}}+\beta_{\mathrm{Net}:\mathrm{Crop}:\mathrm{Season}}\\ & \;+\;\beta_{\mathrm{Crop}:\mathrm{Season}:\mathrm{Block}}+\beta_{\mathrm{Week}}\cdot \text{Week\_z}+\log(\text{n\_thrips\_points})+b_{PlotID}\\ b_{PlotID} & ∼N(0,\;\sigma_{Plot}^{2}) \end{array}$$

#### 2.5.2. Model Selection and Diagnostics

We compared several candidate count models to ensure appropriate fit: NB1, NB2, COM-Poisson, zero-inflated COM-Poisson (ZI COM-Poisson), and zero-inflated negative binomial type 2 (ZINB2). Models were evaluated using AIC, DHARMa (version: 0.4.7) residual diagnostics (uniformity, dispersion, zero-inflation), convergence status, and positive-definiteness of the Hessian matrix. The NB1 model showed the lowest AIC, satisfactory uniformity of residuals, no evidence of overdispersion or zero-inflation, and stable convergence, and was therefore selected as the primary model.

#### 2.5.3. Microclimate Models

For the microclimate models, the response variables were analyzed on their original measurement scales. Z-standardized versions of these variables were created only for use as covariates in the exploratory microclimate-adjusted thrip model:

$$M_{ij}=\gamma_{0}+\gamma_{Net[i]}+\gamma_{Context[i]}+\gamma_{Net\times Context[i]}+\gamma_{w}{\text{Week\_z}}_{ij}+u_{j}+\varepsilon_{ij}$$

where $M_{ij}$ represents the unstandardized 7-day mean temperature, 7-day mean relative humidity, or weekly short-duration illuminance; $u_{j}$ is the plot-level random intercept; and $\varepsilon_{ij}$ is the residual error.

Weekly maximum and minimum hourly temperature and relative humidity were summarized descriptively by net-cage treatment and Crop × Season context. For each treatment–context combination, we report the mean, standard error, and observed plot-week range (Table S8). These supplementary extreme-value summaries were not included as additional covariates in the primary or exploratory microclimate-adjusted thrip models, because they were derived from the same hourly records as the 7-day means and were intended only to broaden the descriptive interpretation of microclimate conditions.

#### 2.5.4. Exploratory Microclimate-Adjusted Thrip Model

To explore whether the measured microclimate variables were associated with variation in the estimated net-cage treatment effects, we refitted the primary NB1 model with the three standardized microclimate variables included as additional covariates:

$$\begin{array}{cc} \log(\mu_{ij})= & \beta_{0}+\beta_{Net[i]}+\beta_{Context[i]}+\beta_{Net\times Context[i]}+\beta_{w}{\text{Week\_z}}_{ij}\\ & +\beta_{T}{\text{Temp\_z}}_{ij}+\beta_{RH}{\text{RH\_z}}_{ij}+\beta_{L}{\text{Light\_z}}_{ij}\\ & +\log({\text{n\_thrips\_points}}_{ij})+b_{j} \end{array}$$

with $b_{j}∼N(0,\sigma_{plot}^{2})$. This model was used solely to assess whether statistical adjustment for microclimate altered the magnitude or precision of the treatment effect estimates. It is an exploratory covariate-adjusted analysis, not a formal causal mediation or effect-decomposition. Any changes in the estimated net-cage treatment effects following adjustment were interpreted only as descriptive changes in the conditional associations. The analysis was not used to estimate mediation, direct or indirect effects, or the proportion of the treatment association attributable to the measured microclimate variables. Because the microclimate variables were not independently manipulated and may represent post-treatment variables, and because unmeasured common causes of microclimate and thrip abundance cannot be excluded, the assumptions required for identification of causal direct and indirect effects were not considered satisfied.

#### 2.5.5. General Diagnostic Procedures

For all mixed models, residual diagnostics were performed with the DHARMa package (version: 0.4.7). Goodness-of-fit was assessed through tests of uniformity, dispersion, and zero-inflation. Statistical significance was set at *p* < 0.05. Model adequacy was evaluated using simulation-based scaled residuals generated with the DHARMa package. We examined residual uniformity using the Kolmogorov–Smirnov test, dispersion, outliers, and residual quantile patterns against model predictions. Diagnostic plots for the primary and microclimate-adjusted NB1 models are presented in Figure S1, and numerical diagnostic comparisons among candidate count distributions are provided in Table S3.

## 3. Results

A total of 324 observations were collected. After excluding 17 observations with fewer than three valid sampling points, 307 observations were retained for the primary analyses. Variation in sampling effort among the retained observations was accounted for using an offset term (Table S2).

### 3.1. Physical Characteristics of Insect-Proof Nets

The material, aperture dimensions, filament diameter and porosity of the three insect-proof nets are summarized in Table 1. All nets were white and made of polyethylene. For each mesh specification, ten 10 cm × 10 cm pieces were measured using optical microscopy and image analysis; porosity was calculated individually for each piece and is reported as mean ± SD.

The 50 measured adult thrips had a mean thorax width of 0.251 ± 0.032 mm and a mean abdomen width of 0.271 ± 0.032 mm (Table S1). Because the specimens were not identified to species, these values characterize the measured adult-thrip sample rather than a particular species. The minimum measured apertures of the 40-, 60-, and 80-mesh nets were 0.922 mm, 0.495 mm, and 0.300 mm, respectively. These values exceeded the mean thorax and abdomen widths of the measured adults. Complete size-based exclusion therefore cannot be inferred from the mean dimensional comparison alone. Smaller apertures may nevertheless impede passage depending on individual size, orientation, behavior, developmental stage, and aperture geometry.

### 3.2. Thrips’ Population Dynamics Under Different Insect-Proof Net-Cage Treatments

**Figure 2:**
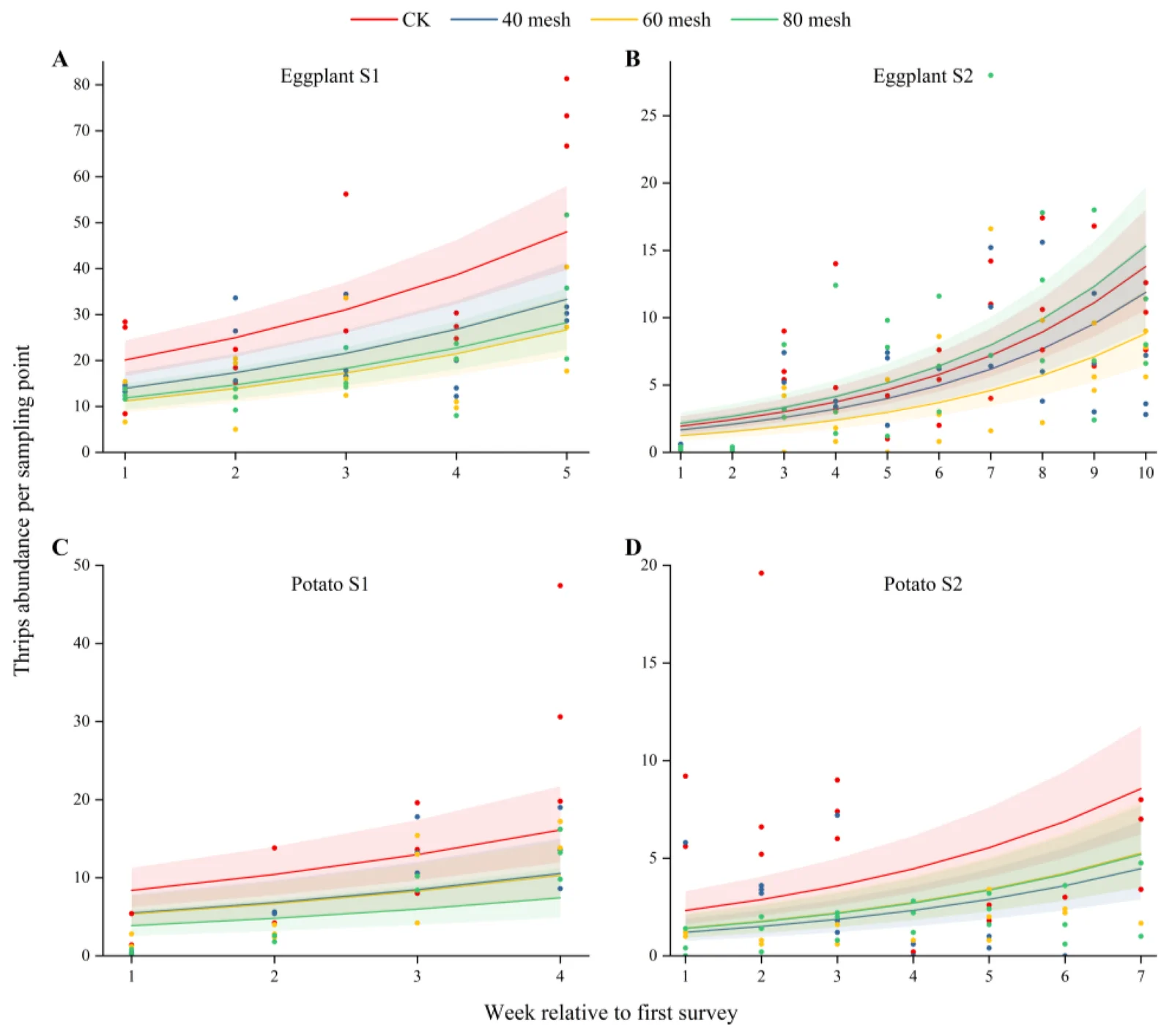
Temporal dynamics of thrip abundance under different insect-proof net-cage treatments across crop–season contexts. Panels show (**A**) Eggplant S1, (**B**) Eggplant S2, (**C**) Potato S1, and (**D**) Potato S2. Points represent observed thrip abundance per sampling point for individual plots at each survey week. Solid lines represent population-level fitted temporal trajectories from the primary NB1 generalized linear mixed model, and shaded ribbons indicate the corresponding 95% confidence intervals. The x-axis represents week relative to the first survey within each crop–season context, and the y-axis represents thrip abundance per sampling point. Panel-specific y-axis ranges are used because absolute thrip abundance differed substantially among crop–season contexts; absolute abundance should therefore be compared using the numerical axis values rather than relative curve height. Formal treatment comparisons were based on the context-specific contrasts from the primary NB1 model reported in Table 2.

We observed that thrip abundance generally increased over time across all four crop–season contexts, although the magnitude of increase differed among contexts (Figure 2). In Eggplant S1, Potato S1, and Potato S2, the fitted trajectories of the three net-cage treatments generally remained below that of CK as the survey progressed. In Eggplant S2, treatment trajectories were less clearly separated, with the 60-mesh treatment tending to show the lowest fitted abundance, whereas the 80-mesh trajectory approached or exceeded that of CK during the later survey period.

Overall, the temporal patterns suggested that net-cage treatments generally reduced thrip abundance relative to CK, but the magnitude and consistency of this pattern varied with crop–season context and mesh size. These treatment differences were subsequently quantified using the primary NB1 generalized linear mixed model.

### 3.3. Context-Specific Effects of Insect-Proof Net on Thrip Abundance

The primary NB1 model showed no evidence of significant residual non-uniformity, dispersion, zero-inflation, outliers, or conditional quantile deviations in the DHARMa diagnostics (Figure S1A and Table S3). The Kolmogorov–Smirnov uniformity test was non-significant (*p* = 0.412), as were the dispersion test (dispersion ratio = 1.178, *p* = 0.207) and zero-inflation test (observed-to-expected zero ratio = 0.746, *p* = 0.299). The outlier test was also non-significant (*p* = 1.000). The estimated PlotID random-intercept variance was 2.88 × 10^−8^ (SD = 1.70 × 10^−4^) and is reported for transparency.

**Figure 3:**
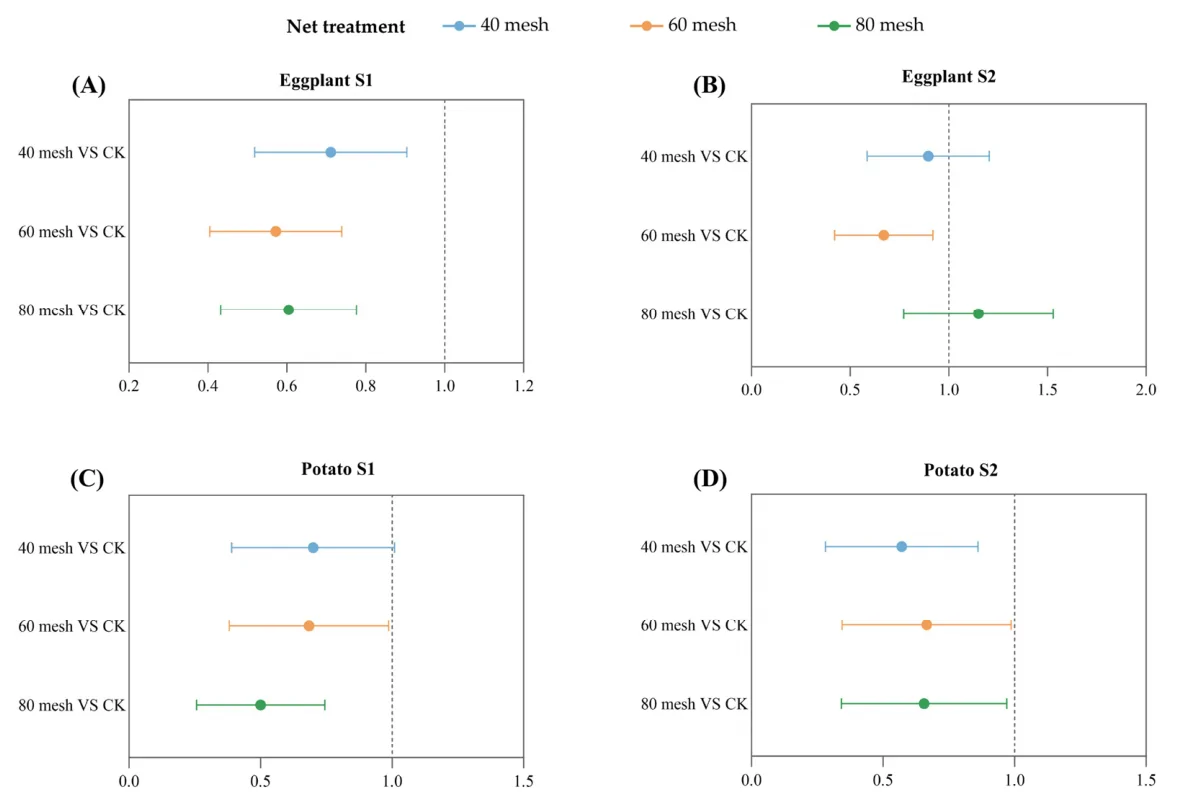
Context-specific net-cage treatment effects on thrip abundance in greenhouse eggplant and potato. Points represent incidence rate ratios (IRRs) of each net-cage treatment relative to CK, and horizontal bars indicate 95% confidence intervals. The vertical dashed line at IRR = 1 indicates no difference in thrips abundance between each net-cage treatment and CK; estimates to the left indicate lower thrips abundance relative to CK, whereas estimates to the right indicate higher thrips abundance. Estimates were obtained from a negative binomial generalized linear mixed model including net-cage treatment, crop species, season, their interactions, relative sampling week, an offset for the number of thrips sampling points, and plot identity as a random effect.

The effects of insect-proof net-cage treatments on thrip abundance varied among crop–season contexts (Table 2 and Figure 3). In Eggplant S1, all three net-cage treatments were associated with lower thrip abundance than CK, corresponding to estimated reductions of 30.6–44.4% (*p* ≤ 0.009). In Eggplant S2, suppression was detected only for the 60-mesh treatment (*p* = 0.020), corresponding to a 36.0% reduction. No detectable suppression was observed for the 40- or 80-mesh treatments in this context.

In Potato S1, the strongest reduction was observed for the 80-mesh treatment (*p* = 0.003), whereas the estimated reductions under 40- and 60-mesh were not statistically supported at the 0.05 level. In Potato S2, the 40- and 80-mesh treatments reduced thrip abundance by 47.8% (*p* = 0.015) and 39.4% (*p* = 0.046), respectively, while the 60-mesh contrast was marginally above the conventional significance threshold (*p* = 0.055).

Overall, the 60-mesh treatment showed the most consistent direction of suppression, with IRRs below 1 in all four contexts and estimated reductions ranging from 36.0% to 44.4%. In contrast, the 80-mesh treatment produced stronger suppression in some contexts but showed no detectable suppression in Eggplant S2. These results indicate that increasing nominal mesh density did not result in a simple monotonic increase in thrip suppression.

Explicit adjustment for the randomized block structure had little influence on the context-specific net-cage treatment estimates. Across the 12 treatment-versus-CK contrasts, all treatment estimates retained the same direction after block adjustment. The median absolute change in IRR was 1.3%, and the maximum change was 2.5% (Table S7). Only one borderline contrast crossed the conventional *p* = 0.05 threshold: for the 60-mesh treatment in potato S1, the estimate changed from IRR = 0.639 (95% CI: 0.406–1.007, *p* = 0.054) in the primary model to IRR = 0.624 (95% CI: 0.397–0.981, *p* = 0.041) after block adjustment. The direction and magnitude of this effect nevertheless remained very similar. Overall, the principal context-dependent treatment pattern was robust to explicit adjustment for the randomized block structure.

### 3.4. Microclimate Differences Associated with Insect-Proof Net-Cage Treatments

**Figure 4:**
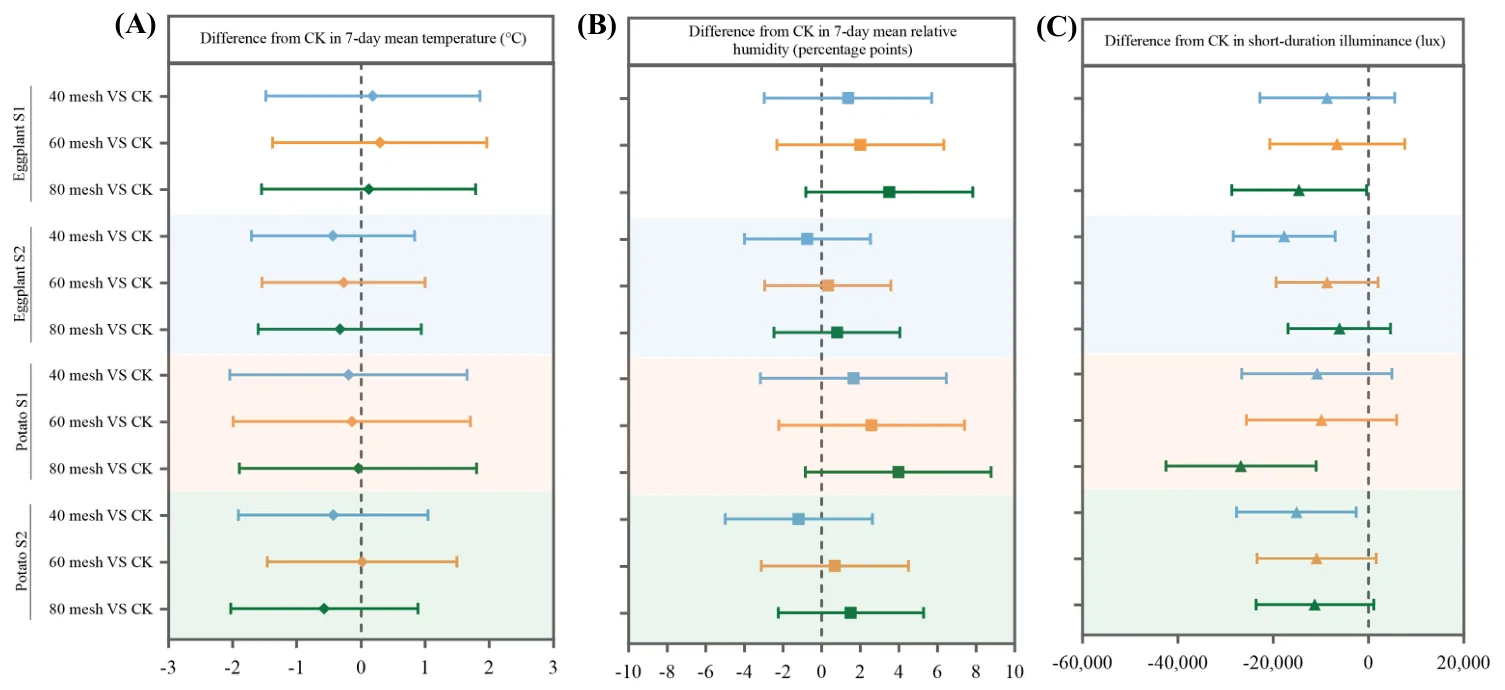
Model-estimated differences in greenhouse microclimate between insect-proof net-cage treatments and the uncovered control (CK). Points represent estimated treatment-minus-CK contrasts for (**A**) 7-day mean temperature, (**B**) 7-day mean relative humidity, and (**C**) weekly short-duration illuminance within each crop–season context; horizontal lines indicate 95% confidence intervals. Blue, orange, and green symbols represent the 40-, 60-, and 80-mesh treatments, respectively. The horizontal background bands visually separate the four Crop × Season contexts, arranged from top to bottom as Eggplant S1, Eggplant S2, Potato S1, and Potato S2; the background shading is used only as a visual guide and has no statistical meaning. The vertical dashed line at zero indicates no difference from CK. Estimates were obtained from repeated-measures linear mixed models with plot identity included as a random intercept. Illuminance represents the mean recorded during an approximately 30 s fixed-time measurement period and should not be interpreted as daily or weekly integrated irradiance.

Microclimate responses to the net-cage treatments differed among environmental variables and crop–season contexts (Figure 4 and Table S5). For 7-day mean temperature, none of the within-context contrasts between the net-cage treatments and CK were statistically significant, indicating no consistent detectable treatment-associated difference in mean temperature relative to CK across the four contexts. Similarly, no statistically supported differences between the net-cage treatments and CK were detected for 7-day mean relative humidity. The largest positive estimates occurred for the 80-mesh treatment in eggplant S1 (+3.51 percentage points) and potato S1 (+3.98 percentage points), but the corresponding 95% confidence intervals included zero.

In contrast, clearer treatment-associated differences were observed for weekly short-duration illuminance, although these effects were context dependent. In S1, illuminance was lower than CK under the 80-mesh treatment in both eggplant (−14,506 lux, 95% CI: −28,693 to −318) and potato (−26,747 lux, 95% CI: −42,498 to −10,996). In S2, significantly lower illuminance was detected under the 40-mesh treatment in eggplant (−17,623 lux, 95% CI: −28,329 to −6918) and potato (−15,099 lux, 95% CI: −27,606 to −2593). The remaining illuminance contrasts were statistically uncertain.

Overall, treatment-associated differences were more evident for short-duration illuminance than for 7-day mean temperature or relative humidity, and the illuminance responses varied among crop–season contexts.

Supplementary descriptive summaries of the hourly records further characterized weekly temperature and relative-humidity extremes (Table S8). Across the 16 treatment–context combinations, mean weekly maximum temperature ranged from 31.38 ± 0.69 °C (40-mesh, Eggplant S2) to 38.23 ± 1.46 °C (80-mesh, Potato S1), whereas mean weekly minimum temperature ranged from 9.83 ± 0.62 °C (80-mesh, Eggplant S2) to 15.01 ± 0.45 °C (CK, Eggplant S1). No consistent monotonic ordering with nominal mesh count was evident for either temperature extreme. Mean weekly maximum relative humidity ranged from 77.21 ± 1.29% (CK, Eggplant S1) to 94.75 ± 0.51% (CK, Potato S2), and mean weekly minimum relative humidity ranged from 20.27 ± 2.72% (CK, Potato S1) to 57.86 ± 2.25% (80-mesh, Eggplant S2). The 60- and 80-mesh treatments had descriptively higher mean weekly minimum relative humidity than CK in all four contexts, but the magnitude varied among crops and seasons. These supplementary summaries did not alter the principal interpretation based on the 7-day mean microclimate models.

### 3.5. Exploratory Sensitivity Analysis of Microclimate Adjustment

**Figure 5:**
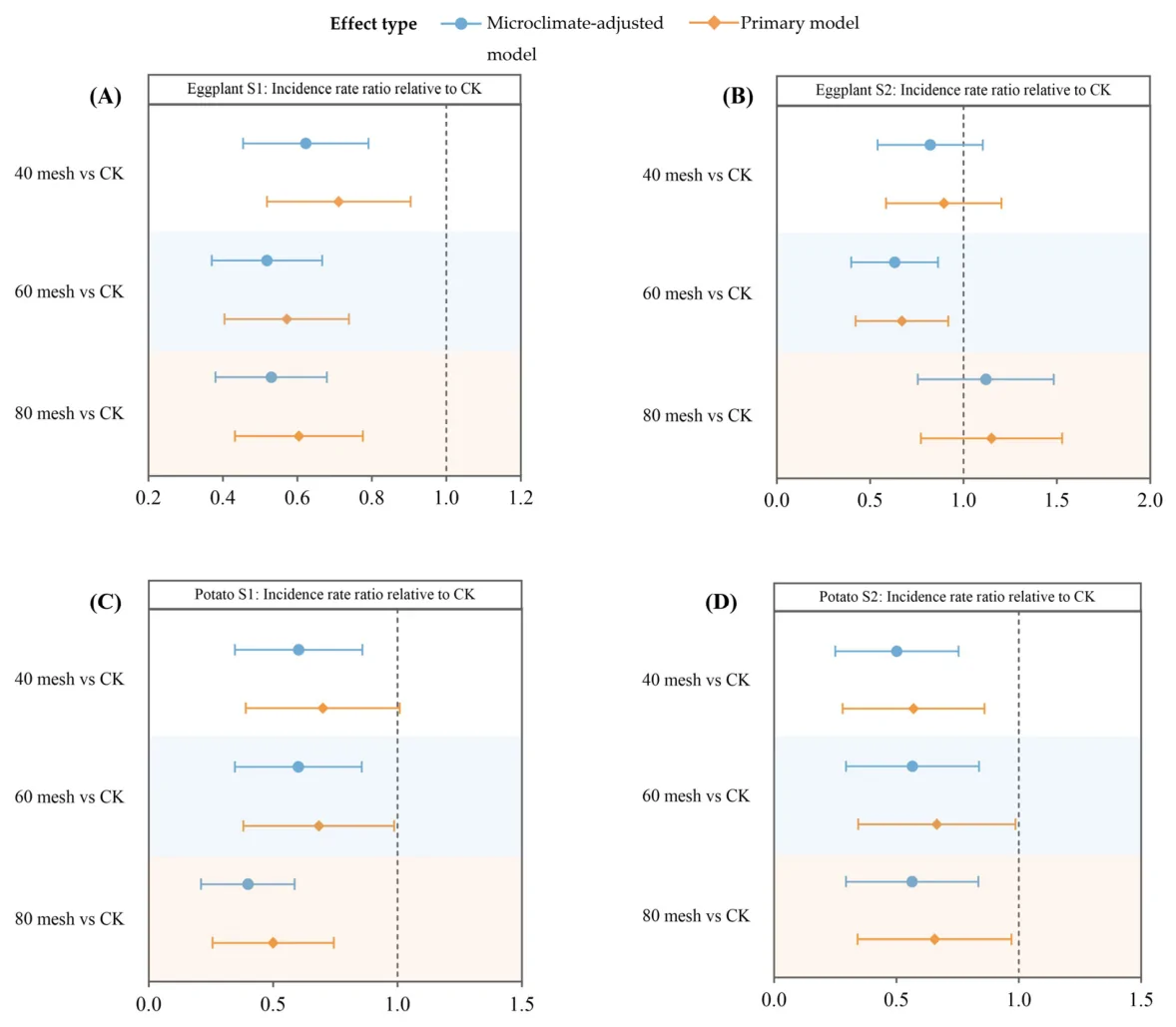
Comparison of context-specific net-cage treatment effects estimated by the primary and microclimate-adjusted NB1 generalized linear mixed models. Points represent incidence rate ratios (IRRs) for each insect-proof net-cage treatment relative to the uncovered control (CK), and horizontal lines indicate 95% confidence intervals. Within each Crop × Season panel, the horizontal background bands visually separate the 40-, 60-, and 80-mesh treatment contrasts from top to bottom; the background shading is used only as a visual guide and has no statistical meaning. The adjusted model additionally included standardized 7-day mean temperature, 7-day mean relative humidity, and weekly short-duration illuminance. Both models were fitted to the same 307 plot-week observations and included sampling effort as an offset and plot identity as a random intercept. The vertical dashed line at IRR = 1 indicates no difference from CK. Differences between the two sets of estimates are presented as an exploratory sensitivity comparison and should not be interpreted as a causal decomposition of treatment effects.

Context-specific IRRs from the primary NB1 model were compared with estimates from an exploratory model additionally adjusted for standardized temperature, relative humidity, and illuminance (Figure 5 and Table S6). The microclimate-adjusted NB1 model likewise showed no significant residual uniformity, dispersion, zero-inflation, outlier, or conditional quantile problems (Figure S1B and Table S3; uniformity test: *p* = 0.455; dispersion ratio = 1.112, *p* = 0.420; zero-inflation ratio = 0.902, *p* = 0.805; outlier test: *p* = 1.000). The estimated PlotID random-intercept variance was 2.24 × 10^−9^ (SD = 4.73 × 10^−5^). Both models were fitted to the same 307 plot-week observations. Adjustment did not consistently attenuate the estimated net-cage effects: most adjusted IRRs remained below 1 and generally shifted farther from the null value, with stronger estimated suppression for several potato contrasts. The 60-mesh treatment retained a suppressive direction in all four contexts, whereas no detectable suppression by the 80-mesh treatment was observed in eggplant S2.

In the adjusted model, temperature was positively associated with thrip abundance (IRR = 1.140, 95% CI: 1.011–1.284, *p* = 0.032), illuminance was negatively associated with abundance (IRR = 0.823, 95% CI: 0.754–0.899, *p* < 0.001), and the relative humidity association was statistically uncertain (IRR = 1.060, 95% CI: 0.878–1.281, *p* = 0.544). These results indicate that the main context-dependent treatment pattern was broadly robust to adjustment for the measured microclimate variables. However, this comparison does not identify mediation or provide a causal decomposition of the net-cage treatment effects.

## 4. Discussion

Across greenhouse eggplant and potato, net-cage treatments generally reduced thrip abundance, but the magnitude and statistical support of suppression depended strongly on crop and season. The principal result was therefore not a simple ranking of mesh sizes. Rather, the 60-mesh treatment showed the most consistent suppressive direction across the four crop–season contexts, whereas the 80-mesh treatment produced the strongest reduction in one context but no detectable suppression in another. Treatment-associated differences in 7-day mean temperature and relative humidity were statistically uncertain, while weekly short-duration illuminance differed from CK in several contexts. Moreover, adjustment for the measured microclimate variables did not attenuate the estimated net-cage effects consistently. Together, these findings support a context-dependent interpretation of net performance and do not establish a causal partition between physical restriction and microclimate-mediated effects.

### 4.1. Context Dependence of Thrips Suppression

The primary NB1 model showed that most treatment to CK incidence rate ratios were below 1, indicating a general tendency toward lower thrip abundance in netted plots. Nevertheless, the estimates varied substantially among the four crop–season contexts. In eggplant S1, all three net-cage treatments reduced thrip abundance, with estimated reductions of 30.6–44.4%. In eggplant S2, only the 60-mesh treatment showed statistically supported suppression (36.0%), whereas the 40-mesh and 80-mesh contrasts were uncertain. In potato S1, the strongest and clearly supported response occurred under the 80-mesh treatment (53.9%), while the 40-mesh and 60-mesh estimates were similar in direction but less precise. In potato S2, suppression was supported for the 40-mesh (47.8%) and 80-mesh (39.4%) treatments, whereas the 60-mesh estimate (38.5%) was marginal (*p* = 0.055). Thus, neither nominal mesh count nor aperture size alone generated a fixed treatment ranking across crops and seasons.

The weekly population trajectories were consistent with this heterogeneity. CK plots generally supported higher thrip abundance, particularly during periods of population increase, but the relative separation among treatments changed over time and among contexts. Such variation is biologically plausible because net-cage performance reflects the balance between immigration into plots, establishment on the host, reproduction within the enclosed crop, and losses from the local population. These processes can vary with seasonal pest pressure, crop phenology, host suitability, canopy architecture, and greenhouse management. Thrips are also highly responsive to host and environmental conditions [21,22,23,24]. Consequently, a net that restricts movement effectively under one crop–season combination may not yield an equivalent reduction when external pest pressure, host condition, or within-cage population growth differs.

Our findings extend earlier evidence that insect-proof screens can contribute to non-chemical pest management in protected cultivation [16,20,25]. They also emphasize that efficacy estimates obtained from one crop or season should not be generalized uncritically. The significant treatment-by-context structure captured by the model is particularly relevant for practical recommendations: performance should be evaluated across representative crops and production periods, rather than inferred from the finest nominal mesh or from a single high-suppression contrast. In this experiment, context dependence was not a secondary source of noise but a central property of the observed treatment response.

### 4.2. Consistent Suppression Direction of the 60-Mesh Treatment

Among the three tested nets, the 60-mesh treatment was the only one with an IRR below 1 in all four contexts. Its estimated reductions occupied a comparatively narrow range of 36.0–44.4%. Statistical support was clear in eggplant S1 and S2, whereas the potato S1 and S2 contrasts were close to, but did not cross, the conventional *p* < 0.05 threshold (*p* = 0.054 and *p* = 0.055). The appropriate interpretation is therefore that 60-mesh showed the most consistent direction and magnitude of estimated suppression, not that it was significantly effective in every context or universally superior to the other nets.

This consistency is agronomically relevant because growers often require a treatment that performs reasonably across variable conditions rather than one that provides the largest reduction only under a particular set of conditions. However, the present design cannot determine why the 60-mesh treatment was more consistent. Its minimum measured aperture remained larger than the adult thorax and abdomen widths, so complete exclusion cannot be inferred from the dimensional comparison. The treatment may instead have restricted entry or movement without preventing passage entirely. Aperture geometry, filament diameter, porosity, installation, access through the zippered opening, and unmeasured airflow may all have influenced the effective barrier.

It would also be premature to attribute the consistency of 60-mesh to an optimal balance between exclusion and microclimate. The 60-mesh and 80-mesh nets had similar calculated porosity despite differing in aperture dimensions and filament diameter, illustrating that nominal mesh count does not summarize all functionally important net properties. Furthermore, the microclimate models did not show a consistently smaller disturbance under 60-mesh than under 80-mesh across all variables and contexts. The present evidence therefore identifies 60-mesh as a promising, comparatively consistent treatment under the tested conditions, while leaving the underlying physical and ecological explanation unresolved.

### 4.3. Context-Dependent Performance of the 80-Mesh Treatment

The 80-mesh treatment illustrates why the finest net should not automatically be regarded as the most effective. It produced substantial and statistically supported reductions in eggplant S1 (41.2%), potato S1 (53.9%), and potato S2 (39.4%), including the largest reduction observed in the primary analysis. In eggplant S2, however, its IRR was 1.109 (95% CI: 0.794–1.548; *p* = 0.544). Because the confidence interval was wide and included 1, this result should be interpreted as no detectable suppression rather than evidence that the 80-mesh treatment increased thrip abundance. This crop–season contrast is an observed statistical pattern. The following explanations are therefore considered hypotheses that may account for the pattern rather than mechanisms demonstrated by the present experiment.

This contrast cannot be readily explained by the measured microclimate variables alone. Treatment-related differences in 7-day mean temperature and relative humidity were generally uncertain, and microclimate adjustment did not consistently attenuate the estimated net-cage effects. The contrasting response of the same 80-mesh treatment among crop–season contexts therefore suggests that crop-specific and seasonal processes may interact with the physical and environmental effects of enclosure.

Eggplant and potato differ in canopy development, leaf arrangement and surface characteristics, phenology, and suitability as hosts for thrips. Such differences could influence several population processes simultaneously. Canopy architecture may affect where immigrating thrips encounter and establish on host tissue, whereas host suitability and crop phenology may influence survival and reproduction after entry. Differences in canopy density may also modify air movement and local leaf-level conditions that were not captured by the plot-level temperature and relative-humidity measurements. Consequently, reduced movement through a finer net does not necessarily translate into proportionally lower population abundance if thrips that enter or are already present can establish and reproduce successfully within a particular crop canopy.

Seasonal pest pressure may further modify these crop-specific responses. Variation in the timing and magnitude of immigration from surrounding vegetation, together with differences in crop developmental stage, could alter the balance between external colonization and within-cage population growth. Management access through the zippered opening may also provide intermittent opportunities for entry. Because external entry pressure, within-cage reproduction, canopy architecture, leaf traits, and host physiological condition were not measured directly, their relative contributions cannot be quantified in the present study. These processes should therefore be regarded as plausible explanations for the observed context dependence rather than demonstrated mechanisms.

### 4.4. Microclimate Associations and Limits of Mechanistic Interpretation

The net-cage treatments did not produce consistent detectable differences in 7-day mean temperature or relative humidity relative to CK. Although some estimated humidity differences were positive, including the largest estimates under 80-mesh in S1, all corresponding within-context confidence intervals included zero. These results differ from a simple expectation that progressively finer nets necessarily increase mean temperature or humidity. Screen effects on greenhouse climate depend on external weather, ventilation configuration, net porosity and pressure drop, crop canopy, irrigation, and sensor position [26,27,28,29,30]. In the present single greenhouse, natural ventilation and the relatively small plot cages may also have limited or varied the average thermal and humidity response.

Mean weekly maximum temperature ranged from 31.38 to 38.23 °C and mean weekly minimum temperature from 9.83 to 15.01 °C across the treatment–context combinations. Temperature extremes showed no consistent monotonic ordering with nominal mesh count. Mean weekly maximum relative humidity ranged from 77.21% to 94.75%, whereas mean weekly minimum relative humidity ranged from 20.27% to 57.86%. The mean weekly minimum relative humidity was descriptively higher under the 60- and 80-mesh treatments than under CK in all four contexts, although these supplementary summaries were not subjected to additional treatment-effect inference and do not establish a uniform causal response to mesh density. Because the loggers were positioned at a fixed height and lacked radiation shields, the absolute daytime temperature maxima should be interpreted especially cautiously, and none of these plot-level summaries necessarily represent the conditions experienced by individual thrips within the crop canopy.

Treatment-associated differences were clearer for weekly short-duration illuminance. Illuminance was lower under 80-mesh in both crops during S1 and under 40-mesh in both crops during S2; the remaining contrasts were statistically uncertain. The absence of a monotonic ordering again suggests that recorded illuminance depended on more than nominal mesh count, potentially including aperture geometry, filament arrangement, solar conditions at the time of measurement, crop canopy, and plot position. Because illuminance was measured for approximately 30 s at a fixed weekly time, it represents a short-duration photometric observation rather than a daily light integral, weekly mean radiation, or photosynthetically active radiation (PAR). Illuminance in lux is weighted according to human visual sensitivity and is not equivalent to PAR, and the ~30 s weekly snapshots do not represent cumulative crop radiation exposure. Plant physiological responses (photosynthetic rate, leaf traits, biomass accumulation, yield) were not measured; the observed illuminance contrasts therefore provide environmental context but cannot quantify altered crop physiological performance. In an exploratory adjusted model, higher standardized temperature was associated with greater thrip abundance, illuminance showed a negative association, and the relative humidity association was uncertain. These conditional associations must be interpreted cautiously: the negative illuminance coefficient may reflect seasonal, crop, canopy, or other correlated variation rather than a direct effect of light on treatment efficacy. Thrip development and behavior can respond to temperature, humidity, and visual cues [23,24,31,32], but the direction and magnitude of such responses in a greenhouse depend on the ranges experienced and on correlations with host condition and time. Adjustment for the three measured microclimate variables did not consistently move the treatment incidence rate ratios (IRRs) toward 1; several adjusted estimates instead indicated stronger suppression. Hence, the measured temperature, relative humidity, and illuminance variables did not explain away the net-cage associations. This comparison is a sensitivity analysis, not a mediation analysis, and its value is descriptive and diagnostic. The persistence of context-specific net-cage effects after adjustment indicates that variation in the measured microclimate variables is insufficient to provide a simple statistical explanation for the observed treatment pattern. This finding narrows the range of plausible simple explanations but does not identify which unmeasured physical, biological, or microclimatic processes generated the remaining treatment associations. Because the covariates were consequences or correlates of treatment and were not independently manipulated—and because unmeasured factors such as airflow, canopy density, pest entry, and plant physiological status could affect both microclimate and thrip abundance—the data do not support numerical partitioning of the observed effect into physical and microclimatic components [33,34]. The most defensible interpretation is that net-cage treatment effects persisted after statistical control for the measured environmental variables, while the causal mechanisms remain unresolved.

### 4.5. Practical Implications, Limitations, and Future Research

From an IPM perspective, the results support further evaluation of insect-proof net-cage treatments as a means of reducing thrip abundance. Under the conditions studied, the 60-mesh treatment warrants particular attention because it maintained a similar suppressive direction across both crops and seasons. This consistency, however, should not be interpreted as evidence that 60-mesh represents an economically or agronomically optimal compromise. The present experiment did not measure net costs, marketable yield, product quality, crop injury, or virus transmission. Mesh selection in commercial production should therefore consider crop and season, expected pest pressure, ventilation requirements, disease risk, crop performance, net durability, labor, and economic feasibility rather than thrips suppression alone. Disease risk and plant physiological consequences were not assessed in this study and should not be inferred from the measured humidity or illuminance alone.

A major design limitation is that CK plots had neither netting nor a steel frame. Thus, CK-versus-treatment contrasts estimate the effect of the complete net-cage system, including the net, frame, zippered access, enclosure, and associated management conditions. They cannot isolate the effect of mesh material itself. This limitation applies particularly to the microclimate contrasts: differences in temperature, relative humidity, or illuminance between CK and net-cage treatments cannot be attributed specifically to the net material because enclosure-related effects were not independently controlled. Although all three netted treatments used frames of the same dimensions, the absence of an open-frame or sham-cage control prevents separation of enclosure effects from net-specific effects. Future experiments should therefore include an open-frame or sham-cage treatment and, where feasible, independently vary aperture or porosity while holding frame structure and other net properties comparable.

The study was conducted in a single greenhouse over two planting seasons and two crop species. Although the repeated-measures design provided four informative crop–season contexts, greenhouse location, crop side, season, and other spatial or operational factors cannot be separated as broadly generalizable sources of variation. Multi-greenhouse and multi-year trials are needed to establish external validity. Measurements of outside thrips pressure and within-cage immigration would also help distinguish barrier performance from local population growth.

Microclimate monitoring imposed additional constraints. Temperature and relative humidity loggers were fixed at 1.5 m, were not adjusted as the crop canopy developed, and were used without radiation shields; radiative effects on readings therefore cannot be excluded. Although the hourly records allowed supplementary calculation of weekly maxima and minima, the measurements did not capture leaf-level conditions or resolve how crop architecture altered the microclimate experienced by thrips. Illuminance was measured only during a short weekly midday interval and in lux rather than as photosynthetically active radiation (PAR) or integrated radiation, and thus represents a short-duration photometric observation rather than cumulative crop light exposure. Air velocity, ventilation rate, leaf-level temperature and humidity, canopy architecture, plant physiological responses, and disease incidence were not measured. Although finer nets may involve different material and management costs and may alter the crop light environment, the present experiment did not quantify net costs, marketable yield, or product quality; a formal cost–benefit comparison among mesh specifications was therefore beyond the scope of this study. Neither physiological consequences of the observed illuminance differences nor disease risk can be inferred from the present microclimate measurements. More intensive environmental monitoring would be required to evaluate the mechanisms by which net construction and crop canopy jointly shape conditions experienced by thrips.

Ventilation management should also be considered together with net selection. The experimental greenhouse relied on natural ventilation through manually adjusted side vents, but vent-opening duration, opening area, and air-exchange rate were not experimentally controlled or incorporated into the statistical models. Consequently, the present study cannot determine whether finer net-cage treatments would require more intensive ventilation management under warm or humid conditions. Changes in greenhouse ventilation could also alter temperature, humidity, and the external immigration pressure of thrips, potentially modifying the observed performance of the net-cage treatments. Future experiments should therefore evaluate net specification and ventilation regime factorially and include direct measurements of airflow or air-exchange rate.

Thrips were not identified to species or developmental stage. The counts may therefore have included taxa or stages with different host preferences, body dimensions, dispersal behavior, and environmental responses. Species- and stage-resolved sampling, together with direct net-penetration tests, would strengthen inference about physical restriction. Because crop injury, marketable yield, product quality, disease incidence, and virus transmission were not directly assessed, reductions in abundance cannot be assumed to translate proportionally into economic benefit or lower tospovirus risk.

Future research should integrate replicated physical characterization of nets, airflow resistance, continuous microclimate and radiation measurements, crop growth and yield, and economic analysis. Experiments that manipulate microclimate independently of net treatment, or use factorial combinations of net geometry and ventilation, would be needed to test mediation or mechanism directly. Until such evidence is available, recommendations should emphasize the observed consistency and context dependence of suppression rather than claim a universal best mesh or a quantified physical-versus-microclimate pathway.

The explicit block-adjusted sensitivity analysis produced treatment estimates that were closely comparable to those of the primary model, indicating that the main context-dependent pattern was not materially altered by adjustment for the randomized block structure.

## 5. Conclusions

Insect-proof net-cage treatments generally suppressed thrip abundance in greenhouse eggplant and potato, but their performance depended strongly on crop and planting season. The results therefore do not support a simple expectation that progressively finer mesh necessarily provides greater suppression. Instead, net performance appears to reflect a context-dependent balance among barrier properties, crop characteristics, seasonal pest pressure, and environmental conditions within the protected system.

Among the tested treatments, the 60-mesh net showed the most consistent suppressive tendency across crop–season contexts, whereas the 80-mesh treatment performed strongly in some contexts but inconsistently in others. This pattern suggests that nominal mesh count alone is insufficient for selecting an insect-proof net for thrip management. The measured differences in mean temperature, relative humidity, and short-duration illuminance did not provide a simple statistical explanation for the observed treatment differences, and the present design does not allow the remaining treatment effects to be attributed specifically to physical restriction or microclimate-mediated mechanisms.

From an IPM perspective, the findings support evaluating insect-proof nets across representative crops and production periods rather than identifying a universally optimal mesh from a single context. The 60-mesh treatment warrants further investigation as a comparatively consistent option under the conditions tested, but practical selection should also account for ventilation, crop performance, disease risk, management requirements, and economic feasibility. Future studies incorporating sham-cage controls, direct measurements of pest immigration and airflow, continuous microclimate monitoring, species-resolved thrip data, and crop and economic outcomes will be necessary to determine the mechanisms underlying context-dependent net performance and to develop more broadly applicable recommendations.

## Figures and Tables

**Table 1:** Measured physical characteristics of the insect-proof nets.

Net-Cage Treatment	Material	Color	Aperture A (mm)	Aperture B (mm)	Filament Diameter (mm)	Porosity (%)
40-mesh	Polyethylene	White	0.944 ± 0.055	0.922 ± 0.014	0.206 ± 0.006	67.03 ± 0.438
60-mesh	Polyethylene	White	0.914 ± 0.030	0.495 ± 0.026	0.190 ± 0.005	59.80 ± 0.672
80-mesh	Polyethylene	White	0.593 ± 0.035	0.300 ± 0.033	0.120 ± 0.005	59.28 ± 2.129

Values are mean ± SD based on ten independent net pieces per mesh. Aperture A and Aperture B denote the horizontal and vertical aperture dimensions, respectively. The measured horizontal filament diameter was used as the representative filament diameter. Porosity was calculated as (A*_i_* × B*_i_*)/[(A*_i_* + d*_i_*) (B*_i_* + d*_i_*)] × 100%.

**Table 2:** Context-specific contrasts of thrip abundance between insect-proof net-cage treatments and the uncovered control (CK), estimated using the primary negative binomial type 1 generalized linear mixed model.

Context	Comparison	SE	z	df	IRR (95% CI)	Reduction (%)	*p*-Value
Eggplant S1	40 vs. CK	0.097	−2.617	∞	0.694 (0.528, 0.912)	30.6	0.009
	60 vs. CK	0.084	−3.897	∞	0.556 (0.413, 0.747)	44.4	<0.001
	80 vs. CK	0.086	−3.618	∞	0.588 (0.441, 0.784)	41.2	<0.001
Eggplant S2	40 vs. CK	0.154	−0.843	∞	0.860 (0.606, 1.221)	14.0	0.399
	60 vs. CK	0.123	−2.318	∞	0.640 (0.439, 0.933)	36.0	0.020
	80 vs. CK	0.189	0.607	∞	1.109 (0.794, 1.548)	−10.9	0.544
Potato S1	40 vs. CK	0.152	−1.831	∞	0.654 (0.416, 1.030)	34.6	0.067
	60 vs. CK	0.148	−1.928	∞	0.639 (0.406, 1.007)	36.1	0.054
	80 vs. CK	0.118	−3.023	∞	0.461 (0.279, 0.762)	53.9	0.003
Potato S2	40 vs. CK	0.140	−2.426	∞	0.522 (0.309, 0.883)	47.8	0.015
	60 vs. CK	0.156	−1.921	∞	0.615 (0.374, 1.010)	38.5	0.055
	80 vs. CK	0.152	−1.991	∞	0.606 (0.370, 0.992)	39.4	0.046

CK, uncovered control; IRR, incidence rate ratio; SE, standard error; CI, confidence interval. SEs are reported on the response (IRR) scale. Tests were based on asymptotic Wald z statistics; therefore, finite denominator degrees of freedom were not applicable (df = ∞). Percentage reduction relative to CK was calculated as (1−IRR) × 100%; a negative value indicates an estimated increase rather than suppression. *p* values are from the prespecified within-context contrasts between each net-cage treatment and CK and were not adjusted for multiple comparisons.

## Data Availability

The data supporting the conclusions of this article are provided within the article.

## Supplementary Materials

The following supporting information can be downloaded at: https://www.mdpi.com/article/10.3390/insects17090883/s1, Figure S1: DHARMa simulation-based residual diagnostics for the primary and exploratory microclimate-adjusted NB1 models; Table S1: Adult thrips’ body-dimension measurements and comparison with net apertures; Table S2: Data structure, sampling effort, missingness, and exclusions; Table S3: Candidate count-distribution comparison and diagnostic summaries; Table S4: Full fixed-effect output of the primary NB1 model; Table S5: Full microclimate linear mixed-model outputs and within-context contrasts; Table S6: Full output and context-specific contrasts of the exploratory microclimate-adjusted NB1 model; Table S7: Sensitivity of context-specific net-cage treatment effects to explicit adjustment for the randomized complete block structure; Table S8: Descriptive summaries of weekly maximum and minimum hourly temperature and relative humidity by net-cage treatment and Crop × Season context.
