# Supplementary material for: Context-Dependent Suppression of Thrips by Insect-Proof Nets in Greenhouse Eggplant and Potato
Source: Insects. 2026 Aug 24;17(9):883. doi: 10.3390/insects17090883 (PMC13607765; doi:10.3390/insects17090883)
Supplement: Supplementary file 1 [file insects-17-00883-s001.zip › supplementary figure.pdf]

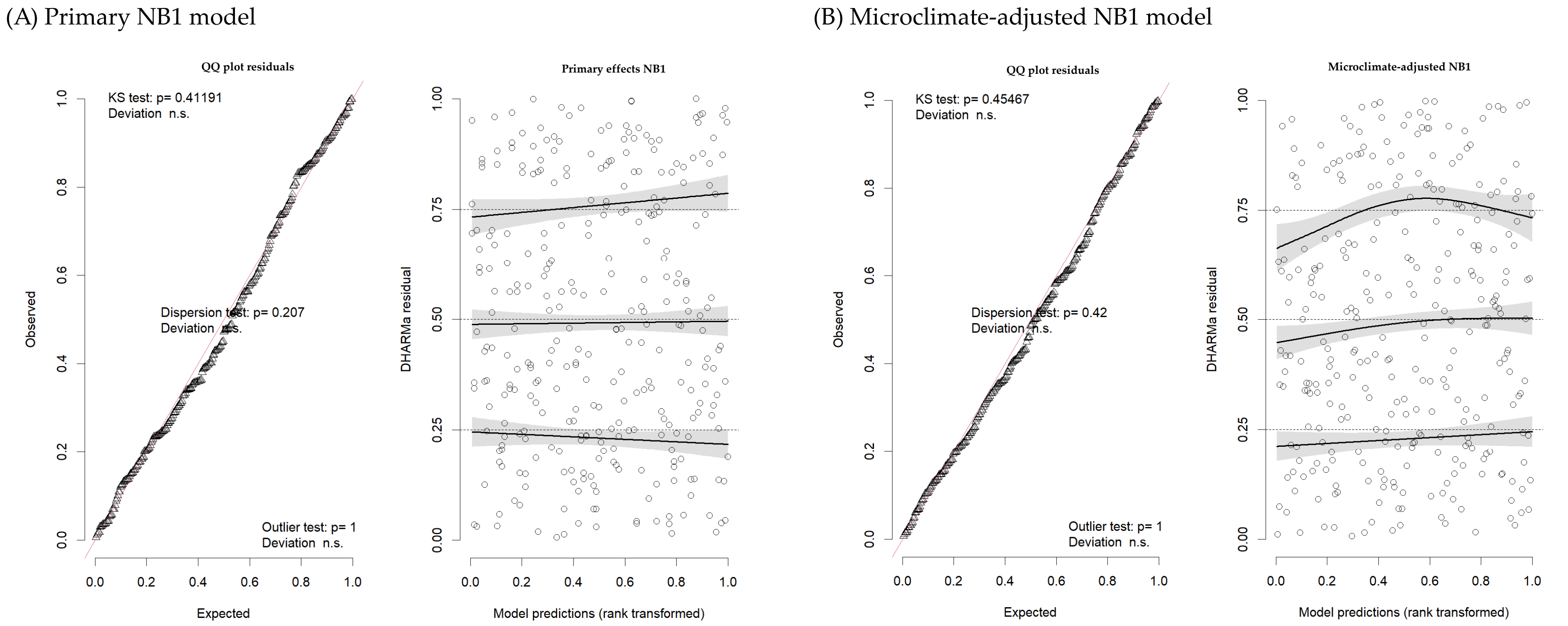

Figure S1. DHARMa simulation-based residual diagnostics for the primary and exploratory microclimate-adjusted NB1 models. (A) Primary NB1 model and (B) exploratory microclimate-adjusted NB1 model. Within each panel, the left plot shows the quantile–quantile plot of simulated scaled residuals together with the Kolmogorov–Smirnov uniformity test, dispersion test, and outlier test. The right plot shows scaled residuals against rank-transformed model predictions, with fitted residual quantile curves. No statistically significant deviations in residual uniformity, dispersion, outliers, or conditional quantiles were detected for either model.
