# Supplementary material for: Context-Dependent Suppression of Thrips by Insect-Proof Nets in Greenhouse Eggplant and Potato
Source: Insects. 2026 Aug 24;17(9):883. doi: 10.3390/insects17090883 (PMC13607765; doi:10.3390/insects17090883)
Supplement: Supplementary file 1 [file insects-17-00883-s001.zip › supplementary tables.pdf]

Table S1. Adult thrips' body-dimension measurements and comparison with net apertures.

Body region	n	Mean width (mm)	Minimum	Maximum
Thorax	50	0.251±0.032	0.162	0.308
Abdomen	50	0.271±0.032	0.173	0.342

Net	Minimum aperture (mm)	Aperture/thorax width ratio	Aperture/abdomen width ratio
40-mesh	0.922	3.67	3.40
60-mesh	0.495	1.97	1.83
80-mesh	0.300	1.20	1.11

* The minimum aperture was defined as the shorter of the two measured aperture dimensions. Ratios greater than 1 indicate that the measured aperture exceeded the corresponding mean adult body width. These dimensional comparisons indicate potential restriction of thrips passage but do not demonstrate complete physical exclusion.

Table S2. Data structure, sampling effort, missingness, and exclusions

(A) **Data structure, sampling effort**

Data category	Number of observations
Total plot-week observations	324
Complete five-point observations	282
Observations with 3-4 valid sampling points	25
Observations with fewer than 3 valid sampling points	17
Observations retained for analysis	307
PlotIDs represented	48

(B) **Exclusion of plot-week observations from the thrip analyses**

Crop	Season	n	unique_PlotID	zeros	zero_percent
eggplant	S1	60	12	0	0
eggplant	S2	120	12	9	7.5
potato	S1	48	12	1	2.083
potato	S2	79	12	3	3.797

Table S3. **Candidate count-distribution comparison and diagnostic summaries**

(A) Candidate count-distribution screening for the primary and microclimate-adjusted thrip models.

Model set	Family	Converged	pdHess	logLik	AIC	Uniformity P	Dispersion ratio	Dispersion P	Zero ratio	Zero-inflation P	PlotID variance	PlotID SD	Status
Primary	NB1	Yes	Yes	−1292.375	2622.750	0.412	1.178	0.207	0.746	0.299	2.88×10^{-8}	1.70×10^{-4}	Pass
Primary	COM-Poisson	Yes	Yes	−1298.180	2634.360	0.504	0.972	0.890	1.272	0.459	1.66×10^{-8}	1.29×10^{-4}	Pass
Primary	ZI COM-Poisson	Yes	Yes	−1298.180	2636.360	0.504	0.977	0.950	1.258	0.458	2.11×10^{-8}	1.45×10^{-4}	Pass
Primary	ZINB2	Yes	Yes	−1315.005	2670.011	0.559	0.495	<0.001	1.569	0.143	2.19×10^{-8}	1.48×10^{-4}	Flagged
Primary	NB2	Yes	Yes	−1315.272	2668.545	0.685	0.474	<0.001	2.068	0.019	1.00×10^{-8}	1.00×10^{-4}	Flagged
Microclimate-adjusted	NB1	Yes	Yes	−1279.610	2603.221	0.455	1.112	0.420	0.902	0.805	2.24×10^{-9}	4.73×10^{-5}	Pass
Microclimate-adjusted	COM-Poisson	Yes	Yes	−1288.303	2620.606	0.531	0.949	0.769	1.340	0.332	1.37×10^{-8}	1.17×10^{-4}	Pass
Microclimate-adjusted	ZI COM-Poisson	Yes	Yes	−1288.303	2622.606	0.337	0.945	0.732	1.331	0.349	2.66×10^{-11}	5.16×10^{-6}	Pass
Microclimate-adjusted	ZINB2	Yes	Yes	−1307.561	2661.123	0.990	0.435	<0.001	1.681	0.101	2.86×10^{-8}	1.69×10^{-4}	Flagged
Microclimate-adjusted	NB2	Yes	Yes	−1307.797	2659.595	0.972	0.416	<0.001	2.243	0.005	1.43×10^{-8}	1.19×10^{-4}	Flagged

(B) NB1 diagnostics for the primary and block-adjusted sensitivity analyses.

Model	Family	Block specification	Converged	pdHess	logLik	AIC	Uniformity P	Dispersion ratio	Dispersion P	Zero ratio	Zero-inflation P	PlotID variance	PlotID SD	Diagnostic status
Primary	NB1	No block term	Yes	Yes	-1292.375	2622.750	0.412	1.178	0.207	0.746	0.299	2.88E-08	1.70E-04	Pass
Primary + block	NB1	Block fixed within Crop × Season	Yes	Yes	-1286.144	2626.288	0.181	1.143	0.297	0.756	0.304	5.47E-10	2.34E-05	Pass
Microclimate-adjusted	NB1	No block term	Yes	Yes	-1279.610	2603.221	0.455	1.112	0.420	0.902	0.805	2.24E-09	4.73E-05	Pass
Microclimate-adjusted + block	NB1	Block fixed within Crop × Season	Yes	Yes	-1273.632	2607.263	0.392	1.070	0.568	0.903	0.821	6.62E-10	2.57E-05	Pass

* Notes: All models used the same 307 plot-week observations. The primary candidate set included Net × Crop × Season, standardized sampling week, the sampling-effort offset, and a PlotID random intercept; the microclimate-adjusted candidate set additionally included standardized 7-day mean temperature, 7-day mean relative humidity, and short-duration illuminance. Panel A was used for distributional screening within each model set. NB1 was retained because it passed DHARMa screening and had the lowest AIC among candidate distributions that passed the diagnostic criteria in both model sets. In Panel B, the block-adjusted models were sensitivity analyses only: randomized block was included as a fixed design-control factor nested within Crop × Season, while PlotID remained the random intercept for repeated weekly observations. Diagnostic status = Pass required successful convergence, a positive-definite Hessian, and DHARMa uniformity, dispersion, and zero-inflation tests with $P \geq 0.05$. AIC values should be compared only among models with the same fixed-effects structure; the block-adjusted models were not used for distribution selection. NB1 = negative binomial type 1; NB2 = negative binomial type 2; ZINB2 = zero-inflated negative binomial type 2; ZI COM-Poisson = zero-inflated Conway–Maxwell–Poisson; pdHess = positive-definite Hessian.

Table S4. Full fixed-effect output of the primary NB1 model.

model_set	family_key	term	estimate	std_error	z_value	p_value	conf_low	conf_high	IRR	IRR_low95	IRR_high95
Primary	NB1	(Intercept)	3.677	0.092	39.984	0.000	3.497	3.857	39.526	33.007	47.333
Primary	NB1	Net40	-0.365	0.140	-2.617	0.009	-0.639	-0.092	0.694	0.528	0.912
Primary	NB1	Net60	-0.588	0.151	-3.897	0.000	-0.883	-0.292	0.556	0.413	0.747
Primary	NB1	Net80	-0.531	0.147	-3.618	0.000	-0.818	-0.243	0.588	0.441	0.784
Primary	NB1	Croppotato	-0.873	0.174	-5.007	0.000	-1.215	-0.532	0.418	0.297	0.588
Primary	NB1	SeasonS2	-2.334	0.168	-13.881	0.000	-2.664	-2.005	0.097	0.070	0.135
Primary	NB1	Week_z	0.542	0.044	12.221	0.000	0.455	0.629	1.719	1.576	1.875
Primary	NB1	Net40:Croppotato	-0.059	0.270	-0.218	0.828	-0.589	0.471	0.943	0.555	1.601
Primary	NB1	Net60:Croppotato	0.141	0.276	0.508	0.611	-0.401	0.682	1.151	0.669	1.978
Primary	NB1	Net80:Croppotato	-0.243	0.295	-0.825	0.409	-0.821	0.335	0.784	0.440	1.398
Primary	NB1	Net40:SeasonS2	0.214	0.226	0.947	0.344	-0.229	0.658	1.239	0.795	1.932
Primary	NB1	Net60:SeasonS2	0.141	0.245	0.577	0.564	-0.338	0.620	1.152	0.713	1.860
Primary	NB1	Net80:SeasonS2	0.634	0.225	2.823	0.005	0.194	1.074	1.885	1.214	2.928
Primary	NB1	Croppotato:SeasonS2	1.048	0.266	3.942	0.000	0.527	1.570	2.853	1.694	4.805
Primary	NB1	Net40:Croppotato:SeasonS2	-0.441	0.420	-1.048	0.294	-1.264	0.383	0.644	0.282	1.467
Primary	NB1	Net60:Croppotato:SeasonS2	-0.181	0.421	-0.430	0.667	-1.006	0.644	0.835	0.366	1.905
Primary	NB1	Net80:Croppotato:SeasonS2	-0.360	0.423	-0.852	0.394	-1.190	0.469	0.697	0.304	1.598

Table S5. Full microclimate linear mixed-model outputs and within-context contrasts.

(A) Fixed-effect coefficients

response	term	estimate	std_error	df	t_value	p-value	lower95	upper95
Temperature	(Intercept)	22.635	0.593	70.852	38.175	0.000	21.453	23.817
Temperature	Net40	0.187	0.833	69.339	0.224	0.823	-1.476	1.849
Temperature	Net60	0.294	0.833	69.339	0.353	0.725	-1.368	1.956
Temperature	Net80	0.123	0.833	69.339	0.147	0.883	-1.540	1.785
Temperature	Croppotato	0.331	0.883	84.755	0.375	0.709	-1.425	2.087
Temperature	SeasonS2	-5.222	0.739	44.629	-7.062	0.000	-6.712	-3.732
Temperature	Week_z	-1.175	0.147	268.387	-7.986	0.000	-1.465	-0.885
Temperature	Net40:Croppotato	-0.378	1.249	84.596	-0.303	0.763	-2.861	2.104
Temperature	Net60:Croppotato	-0.436	1.249	84.596	-0.349	0.728	-2.918	2.047
Temperature	Net80:Croppotato	-0.164	1.249	84.596	-0.132	0.896	-2.647	2.318
Temperature	Net40:SeasonS2	-0.623	1.025	41.294	-0.608	0.547	-2.692	1.446
Temperature	Net60:SeasonS2	-0.563	1.025	41.294	-0.550	0.586	-2.632	1.506
Temperature	Net80:SeasonS2	-0.452	1.025	41.294	-0.441	0.661	-2.521	1.617
Temperature	Croppotato:SeasonS2	3.746	1.100	54.055	3.404	0.001	1.539	5.952
Temperature	Net40:Croppotato:SeasonS2	0.384	1.562	54.772	0.246	0.807	-2.747	3.515
Temperature	Net60:Croppotato:SeasonS2	0.726	1.562	54.772	0.465	0.644	-2.405	3.857
Temperature	Net80:Croppotato:SeasonS2	-0.078	1.558	54.213	-0.050	0.960	-3.201	3.046
Relative_humidity	(Intercept)	53.398	1.539	290.000	34.700	0.000	50.370	56.427
Relative_humidity	Net40	1.377	2.163	290.000	0.637	0.525	-2.879	5.634
Relative_humidity	Net60	2.010	2.163	290.000	0.929	0.353	-2.246	6.266
Relative_humidity	Net80	3.511	2.163	290.000	1.624	0.106	-0.745	7.768
Relative_humidity	Croppotato	-0.264	2.295	290.000	-0.115	0.908	-4.782	4.253
Relative_humidity	SeasonS2	29.931	1.913	290.000	15.649	0.000	26.167	33.695
Relative_humidity	Week_z	-0.025	0.386	290.000	-0.064	0.949	-0.785	0.735
Relative_humidity	Net40:Croppotato	0.273	3.244	290.000	0.084	0.933	-6.112	6.657
Relative_humidity	Net60:Croppotato	0.590	3.244	290.000	0.182	0.856	-5.795	6.975
Relative_humidity	Net80:Croppotato	0.464	3.244	290.000	0.143	0.886	-5.921	6.848
Relative_humidity	Net40:SeasonS2	-2.110	2.649	290.000	-0.797	0.426	-7.323	3.103
Relative_humidity	Net60:SeasonS2	-1.682	2.649	290.000	-0.635	0.526	-6.895	3.531
Relative_humidity	Net80:SeasonS2	-2.708	2.649	290.000	-1.022	0.307	-7.921	2.505

Relative_humidity	Croppotato:SeasonS2	-11.854	2.851	290.000	-4.159	0.000	-17.464	-6.244
Relative_humidity	Net40:Croppotato:SeasonS2	-0.716	4.047	290.000	-0.177	0.860	-8.682	7.250
Relative_humidity	Net60:Croppotato:SeasonS2	-0.225	4.047	290.000	-0.056	0.956	-8.191	7.741
Relative_humidity	Net80:Croppotato:SeasonS2	0.256	4.036	290.000	0.063	0.949	-7.687	8.199
Light	(Intercept)	56762.416	5042.758	290.000	11.256	0.000	46837.370	66687.461
Light	Net40	-8605.493	7086.926	290.000	-1.214	0.226	-22553.824	5342.837
Light	Net60	-6506.907	7086.926	290.000	-0.918	0.359	-20455.237	7441.424
Light	Net80	-14505.533	7086.926	290.000	-2.047	0.042	-28453.864	-557.203
Light	Croppotato	26311.512	7521.119	290.000	3.498	0.001	11508.613	41114.412
Light	SeasonS2	761.882	6267.734	290.000	0.122	0.903	-11574.133	13097.898
Light	Week_z	-9735.872	1265.582	290.000	-7.693	0.000	-12226.762	-7244.982
Light	Net40:Croppotato	-2148.307	10630.389	290.000	-0.202	0.840	-23070.803	18774.189
Light	Net60:Croppotato	-3300.893	10630.389	290.000	-0.311	0.756	-24223.389	17621.603
Light	Net80:Croppotato	-12241.108	10630.389	290.000	-1.152	0.250	-33163.604	8681.388
Light	Net40:SeasonS2	-9017.954	8679.676	290.000	-1.039	0.300	-26101.101	8065.192
Light	Net60:SeasonS2	-2139.173	8679.676	290.000	-0.246	0.806	-19222.320	14943.973
Light	Net80:SeasonS2	8410.040	8679.676	290.000	0.969	0.333	-8673.106	25493.187
Light	Croppotato:SeasonS2	-41058.215	9341.018	290.000	-4.395	0.000	-59443.000	-22673.430
Light	Net40:Croppotato:SeasonS2	4672.309	13262.956	290.000	0.352	0.725	-21431.548	30776.166
Light	Net60:Croppotato:SeasonS2	1131.526	13262.956	290.000	0.085	0.932	-24972.331	27235.383
Light	Net80:Croppotato:SeasonS2	7126.934	13224.778	290.000	0.539	0.590	-18901.782	33155.650

(B) Within-context treatment-minus-CK contrasts

response	Crop	Season	contrast	estimate_difference	SE	df	lower95	upper95	t_value	p_value
Temperature	eggplant	S1	40 - CK	0.187	0.833	56.334	-1.483	1.856	0.224	0.824
Temperature	eggplant	S1	60 - CK	0.294	0.833	56.334	-1.375	1.963	0.353	0.726
Temperature	eggplant	S1	80 - CK	0.123	0.833	56.334	-1.547	1.792	0.147	0.884
Temperature	potato	S1	40 - CK	-0.192	0.930	83.553	-2.041	1.657	-0.206	0.837
Temperature	potato	S1	60 - CK	-0.142	0.930	83.553	-1.991	1.707	-0.152	0.879
Temperature	potato	S1	80 - CK	-0.042	0.930	83.553	-1.891	1.807	-0.045	0.964
Temperature	eggplant	S2	40 - CK	-0.436	0.596	14.893	-1.707	0.835	-0.732	0.476
Temperature	eggplant	S2	60 - CK	-0.269	0.596	14.893	-1.540	1.002	-0.452	0.658
Temperature	eggplant	S2	80 - CK	-0.330	0.596	14.893	-1.601	0.942	-0.553	0.589
Temperature	potato	S2	40 - CK	-0.430	0.726	32.800	-1.907	1.047	-0.593	0.557
Temperature	potato	S2	60 - CK	0.021	0.726	32.800	-1.456	1.498	0.030	0.977
Temperature	potato	S2	80 - CK	-0.571	0.716	31.148	-2.032	0.889	-0.798	0.431
Relative_humidity	eggplant	S1	40 - CK	1.377	2.163	57.756	-2.952	5.707	0.637	0.527
Relative_humidity	eggplant	S1	60 - CK	2.010	2.163	57.756	-2.319	6.339	0.929	0.357
Relative_humidity	eggplant	S1	80 - CK	3.511	2.163	57.756	-0.818	7.841	1.624	0.110
Relative_humidity	potato	S1	40 - CK	1.650	2.418	86.143	-3.157	6.457	0.682	0.497
Relative_humidity	potato	S1	60 - CK	2.600	2.418	86.143	-2.207	7.407	1.075	0.285
Relative_humidity	potato	S1	80 - CK	3.975	2.418	86.143	-0.832	8.782	1.644	0.104
Relative_humidity	eggplant	S2	40 - CK	-0.733	1.529	14.621	-3.999	2.534	-0.479	0.639
Relative_humidity	eggplant	S2	60 - CK	0.328	1.529	14.621	-2.939	3.595	0.215	0.833
Relative_humidity	eggplant	S2	80 - CK	0.803	1.529	14.621	-2.464	4.070	0.525	0.607
Relative_humidity	potato	S2	40 - CK	-1.176	1.876	33.174	-4.992	2.641	-0.627	0.535
Relative_humidity	potato	S2	60 - CK	0.693	1.876	33.174	-3.124	4.509	0.369	0.714
Relative_humidity	potato	S2	80 - CK	1.523	1.851	31.455	-2.250	5.296	0.823	0.417
Light	eggplant	S1	40 - CK	-8605.493	7086.926	57.756	-22792.792	5581.805	-1.214	0.230
Light	eggplant	S1	60 - CK	-6506.907	7086.926	57.756	-20694.205	7680.392	-0.918	0.362
Light	eggplant	S1	80 - CK	-14505.533	7086.926	57.756	-28692.832	-318.235	-2.047	0.045
Light	potato	S1	40 - CK	-10753.800	7923.424	86.143	-26504.674	4997.074	-1.357	0.178
Light	potato	S1	60 - CK	-9807.800	7923.424	86.143	-25558.674	5943.074	-1.238	0.219
Light	potato	S1	80 - CK	-26746.642	7923.424	86.143	-42497.515	-10995.768	-3.376	0.001
Light	eggplant	S2	40 - CK	-17623.448	5011.213	14.621	-28328.764	-6918.132	-3.517	0.003
Light	eggplant	S2	60 - CK	-8646.080	5011.213	14.621	-19351.396	2059.236	-1.725	0.106

(B) Within-context treatment-minus-CK contrasts

response	Crop	Season	contrast	estimate_difference	SE	df	lower95	upper95	t_value	p_value
Light	eggplant	S2	80 - CK	-6095.493	5011.213	14.621	-16800.809	4609.823	-1.216	0.243
Light	potato	S2	40 - CK	-15099.445	6148.400	33.174	-27605.968	-2592.923	-2.456	0.019
Light	potato	S2	60 - CK	-10815.447	6148.400	33.174	-23321.970	1691.075	-1.759	0.088
Light	potato	S2	80 - CK	-11209.667	6065.425	31.455	-23572.933	1153.598	-1.848	0.074

Table S6. Full output and context-specific contrasts of the exploratory microclimate-adjusted NB1 model.

(A). Fixed-effect coefficients

model_set	family_key	term	estimate	std_error	z_value	p_value	conf_low	conf_high	IRR	IRR_low95	IRR_high95
Microclimate-adjusted	NB1	(Intercept)	3.705	0.154	24.137	0.000	3.404	4.006	40.654	30.092	54.925
Microclimate-adjusted	NB1	Net40	-0.498	0.139	-3.584	0.000	-0.770	-0.226	0.608	0.463	0.798
Microclimate-adjusted	NB1	Net60	-0.684	0.147	-4.642	0.000	-0.973	-0.395	0.505	0.378	0.674
Microclimate-adjusted	NB1	Net80	-0.662	0.146	-4.545	0.000	-0.947	-0.376	0.516	0.388	0.686
Microclimate-adjusted	NB1	Croppotato	-0.678	0.168	-4.033	0.000	-1.007	-0.348	0.508	0.365	0.706
Microclimate-adjusted	NB1	SeasonS2	-2.247	0.268	-8.370	0.000	-2.774	-1.721	0.106	0.062	0.179
Microclimate-adjusted	NB1	Week_z	0.470	0.049	9.617	0.000	0.374	0.566	1.600	1.454	1.761
Microclimate-adjusted	NB1	Temp_z	0.131	0.061	2.142	0.032	0.011	0.250	1.140	1.011	1.284
Microclimate-adjusted	NB1	RH_z	0.058	0.096	0.606	0.544	-0.131	0.247	1.060	0.878	1.281
Microclimate-adjusted	NB1	Light_z	-0.195	0.045	-4.339	0.000	-0.283	-0.107	0.823	0.754	0.899
Microclimate-adjusted	NB1	Net40:Croppotato	-0.070	0.259	-0.272	0.786	-0.578	0.437	0.932	0.561	1.548
Microclimate-adjusted	NB1	Net60:Croppotato	0.114	0.263	0.434	0.664	-0.402	0.630	1.121	0.669	1.878
Microclimate-adjusted	NB1	Net80:Croppotato	-0.335	0.280	-1.194	0.233	-0.884	0.215	0.716	0.413	1.240
Microclimate-adjusted	NB1	Net40:SeasonS2	0.262	0.222	1.178	0.239	-0.174	0.698	1.299	0.840	2.009
Microclimate-adjusted	NB1	Net60:SeasonS2	0.180	0.240	0.749	0.454	-0.290	0.649	1.197	0.748	1.914
Microclimate-adjusted	NB1	Net80:SeasonS2	0.739	0.220	3.367	0.001	0.309	1.170	2.095	1.362	3.221
Microclimate-adjusted	NB1	Croppotato:SeasonS2	0.711	0.273	2.600	0.009	0.175	1.246	2.035	1.191	3.478
Microclimate-adjusted	NB1	Net40:Croppotato:SeasonS2	-0.469	0.407	-1.152	0.249	-1.267	0.329	0.626	0.282	1.390
Microclimate-adjusted	NB1	Net60:Croppotato:SeasonS2	-0.259	0.409	-0.632	0.527	-1.061	0.543	0.772	0.346	1.722
Microclimate-adjusted	NB1	Net80:Croppotato:SeasonS2	-0.394	0.409	-0.963	0.336	-1.196	0.408	0.674	0.302	1.504

(B). Context-specific adjusted treatment-versus-CK contrasts

Context	Treatment	IRR	SE	Lower 95% CI	Upper 95% CI	z	P	Reduction (%)
Eggplant S1	40-mesh	0.607832599	0.084428755	0.462967989	0.798025948	-3.584240509	0.00033806	39.2167401
Eggplant S1	60-mesh	0.504657709	0.074344604	0.378094663	0.673586346	-4.642202835	3.44714E-06	49.53422915
Eggplant S1	80-mesh	0.515955295	0.075115296	0.387873393	0.686331859	-4.545355949	5.48425E-06	48.40447045
Eggplant S2	40-mesh	0.78972906	0.140129469	0.557751796	1.118189116	-1.3303959	0.183387865	21.02709399
Eggplant S2	60-mesh	0.60390449	0.115191308	0.4155339	0.87766758	-2.644059935	0.008191815	39.60955105
Eggplant S2	80-mesh	1.080701083	0.181822207	0.777133187	1.502850283	0.461292337	0.644588882	-8.070108295
Potato S1	40-mesh	0.566500892	0.125857726	0.366515058	0.875607301	-2.557882025	0.010531181	43.34991082
Potato S1	60-mesh	0.565777496	0.1251971	0.366681501	0.872976068	-2.573870002	0.010056805	43.42225037
Potato S1	80-mesh	0.369212602	0.091420004	0.227254336	0.599847498	-4.024032054	5.72101E-05	63.07873984
Potato S2	40-mesh	0.460506815	0.121429464	0.274653819	0.772122983	-2.940717129	0.003274534	53.94931854
Potato S2	60-mesh	0.522738439	0.13192038	0.318765846	0.857229463	-2.570390298	0.010158399	47.72615614
Potato S2	80-mesh	0.521515987	0.131562139	0.318079847	0.855064938	-2.580642996	0.009861651	47.84840132

Table S7. Sensitivity of context-specific net-cage treatment effects to explicit adjustment for the randomized complete block structure.

Context	Comparison	Primary model IRR (95% CI)	Primary reduction (%)	P	Block-adjusted IRR (95% CI)	Block-adjusted reduction (%)	P	Change in IRR (%)
Eggplant S1	40 vs CK	0.694 (0.528-0.912)	30.6	0.009	0.679 (0.519-0.888)	32.1	0.005	−2.2
Eggplant S1	60 vs CK	0.556 (0.413-0.747)	44.4	<0.001	0.557 (0.417-0.743)	44.3	<0.001	+0.2
Eggplant S1	80 vs CK	0.588 (0.441-0.784)	41.2	<0.001	0.582 (0.439-0.772)	41.8	<0.001	−1.0
Eggplant S2	40 vs CK	0.860 (0.606-1.221)	14.0	0.399	0.870 (0.615-1.232)	13.0	0.434	+1.2
Eggplant S2	60 vs CK	0.640 (0.439-0.933)	36.0	0.020	0.649 (0.447-0.942)	35.1	0.023	+1.4
Eggplant S2	80 vs CK	1.109 (0.794-1.548)	−10.9	0.544	1.127 (0.809-1.571)	−12.7	0.479	+1.7
Potato S1	40 vs CK	0.654 (0.416-1.030)	34.6	0.067	0.642 (0.409-1.006)	35.8	0.053	−1.9
**Potato S1**	**60 vs CK**	**0.639 (0.406-1.007)**	**36.1**	**0.054**	**0.624 (0.397-0.981)**	**37.6**	**0.041**	**−2.4**
Potato S1	80 vs CK	0.461 (0.279-0.762)	53.9	0.003	0.449 (0.273-0.740)	55.1	0.002	−2.5
Potato S2	40 vs CK	0.522 (0.309-0.883)	47.8	0.015	0.522 (0.309-0.881)	47.8	0.015	0.0
Potato S2	60 vs CK	0.615 (0.374-1.010)	38.5	0.055	0.612 (0.373-1.002)	38.8	0.051	−0.5
Potato S2	80 vs CK	0.606 (0.370-0.992)	39.4	0.046	0.605 (0.371-0.987)	39.5	0.044	−0.2

* The primary model was a negative binomial type 1 generalized linear mixed model (NB1 GLMM) including Net × Crop × Season, standardized sampling week, an offset for sampling effort, and a PlotID random intercept. For the block-adjusted sensitivity analysis, randomized block was additionally included as a fixed design-control factor nested within Crop × Season, while PlotID remained the random intercept for repeated weekly observations. IRR < 1 indicates lower thrips abundance relative to CK. Reduction (%) was calculated as (1 − IRR) × 100; negative values indicate higher estimated abundance relative to CK. Change in IRR (%) was calculated as 100 × (IRR_block-adjusted − IRR_primary)/IRR_primary. Planned Net-versus-CK contrasts were not adjusted for multiplicity.

Table S8. Descriptive summaries of weekly maximum and minimum hourly temperature and relative humidity by net-cage treatment and Crop × Season context.

Context	Net	Number of plot-week observations	Weekly maximum temperature (℃)	Weekly minimum temperature (℃)	Weekly maximum relative humidity (%)	Weekly minimum relative humidity (%)
Eggplant S1	CK	18	36.06 ± 0.77 (30.50-42.00)	15.01 ± 0.45 (11.85-17.95)	77.21 ± 1.29 (63.20-85.05)	23.98 ± 2.02 (14.50-40.55)
Eggplant S1	40-mesh	18	37.11 ± 0.78 (30.50-42.15)	14.86 ± 0.43 (11.75-17.80)	79.21 ± 1.31 (64.85-87.00)	24.40 ± 1.72 (15.00-40.00)
Eggplant S1	60-mesh	18	37.67 ± 0.70 (31.45-42.80)	14.91 ± 0.42 (11.70-17.80)	79.49 ± 1.14 (67.40-86.50)	24.12 ± 1.73 (13.00-39.35)
Eggplant S1	80-mesh	18	36.83 ± 0.68 (31.65-41.60)	14.91 ± 0.42 (11.60-17.75)	81.04 ± 1.04 (69.85-87.25)	26.35 ± 1.99 (15.10-42.20)
Eggplant S2	CK	30	32.43 ± 0.72 (23.90-39.20)	9.89 ± 0.62 (4.60-15.15)	94.19 ± 0.34 (90.35-98.70)	54.30 ± 2.28 (32.90-79.00)
Eggplant S2	40-mesh	30	31.38 ± 0.69 (22.85-37.95)	9.85 ± 0.62 (4.50-15.20)	92.97 ± 0.52 (87.55-97.85)	54.72 ± 2.40 (28.40-75.15)
Eggplant S2	60-mesh	30	31.75 ± 0.68 (23.30-38.30)	9.90 ± 0.62 (4.55-15.35)	94.26 ± 0.39 (90.20-98.35)	55.84 ± 2.28 (31.65-75.20)
Eggplant S2	80-mesh	30	31.48 ± 0.69 (22.00-37.70)	9.83 ± 0.62 (4.65-15.15)	94.35 ± 0.38 (90.20-98.40)	57.86 ± 2.25 (32.10-76.25)
Potato S1	CK	12	37.12 ± 1.38 (29.10-43.10)	14.53 ± 0.63 (11.80-18.60)	80.06 ± 1.11 (74.60-86.30)	20.27 ± 2.72 (12.70-44.60)
Potato S1	40-mesh	12	37.58 ± 1.45 (29.00-43.40)	14.16 ± 0.61 (11.60-18.00)	80.29 ± 1.16 (74.60-86.60)	22.57 ± 2.69 (15.00-46.80)
Potato S1	60-mesh	12	37.75 ± 1.47 (28.90-43.30)	14.11 ± 0.60 (11.70-17.90)	81.52 ± 0.96 (76.50-86.70)	22.88 ± 2.64 (14.20-45.60)
Potato S1	80-mesh	12	38.23 ± 1.46 (28.90-44.00)	14.15 ± 0.59 (11.80-17.70)	82.36 ± 0.86 (78.80-87.30)	24.50 ± 2.69 (16.20-46.80)
Potato S2	CK	21	33.42 ± 0.79 (27.60-37.80)	9.66 ± 0.58 (5.25-14.75)	94.75 ± 0.51 (91.70-99.61)	47.67 ± 2.48 (31.45-73.00)
Potato S2	40-mesh	21	32.12 ± 0.70 (25.75-37.40)	10.05 ± 0.60 (5.10-14.55)	91.56 ± 0.52 (86.40-96.40)	48.62 ± 2.77 (29.05-74.00)
Potato S2	60-mesh	21	32.68 ± 0.67 (27.25-38.50)	10.41 ± 0.60 (5.65-14.95)	92.43 ± 0.40 (89.10-96.45)	51.17 ± 2.28 (35.00-71.75)
Potato S2	80-mesh	21	31.86 ± 0.57 (27.00-35.70)	10.70 ± 0.69 (5.10-17.80)	92.83 ± 0.34 (89.80-96.60)	52.61 ± 2.48 (33.05-74.25)

* Values are means ± standard errors across plot-week observations. A plot-week represents one weekly microclimate summary from one experimental plot. Each treatment had three replicate plots within each Crop × Season context, with repeated weekly observations obtained from the same plots. CK denotes the uncovered and unframed control. Weekly maxima and minima were calculated within the same 7-day windows used for the mean microclimate variables. These summaries are descriptive and were not included as additional covariates in the primary or exploratory microclimate-adjusted thrips models.
